# Complement System Part I – Molecular Mechanisms of Activation and Regulation

**DOI:** 10.3389/fimmu.2015.00262

**Published:** 2015-06-02

**Authors:** Nicolas S. Merle, Sarah Elizabeth Church, Veronique Fremeaux-Bacchi, Lubka T. Roumenina

**Affiliations:** ^1^UMR_S 1138, Cordeliers Research Center, Complement and Diseases Team, INSERM, Paris, France; ^2^UMR_S 1138, Centre de Recherche des Cordeliers, Sorbonne Paris Cité, Université Paris Descartes, Paris, France; ^3^UMR_S 1138, Centre de Recherche des Cordeliers, Sorbonne Universités, Université Pierre et Marie Curie-Paris, Paris, France; ^4^UMR_S 1138, Cordeliers Research Center, Integrative Cancer Immunology Team, INSERM, Paris, France; ^5^Service d’Immunologie Biologique, Assistance Publique-Hôpitaux de Paris, Hôpital Européen Georges-Pompidou, Paris, France

**Keywords:** complement system proteins, complement regulatory proteins, structure–function relationships, anaphylatoxins, membrane-attack-complex, classical complement pathway, alternative complement pathway, endothelial cells

## Abstract

Complement is a complex innate immune surveillance system, playing a key role in defense against pathogens and in host homeostasis. The complement system is initiated by conformational changes in recognition molecular complexes upon sensing danger signals. The subsequent cascade of enzymatic reactions is tightly regulated to assure that complement is activated only at specific locations requiring defense against pathogens, thus avoiding host tissue damage. Here, we discuss the recent advances describing the molecular and structural basis of activation and regulation of the complement pathways and their implication on physiology and pathology. This article will review the mechanisms of activation of alternative, classical, and lectin pathways, the formation of C3 and C5 convertases, the action of anaphylatoxins, and the membrane-attack-complex. We will also discuss the importance of structure–function relationships using the example of atypical hemolytic uremic syndrome. Lastly, we will discuss the development and benefits of therapies using complement inhibitors.

## Introduction

**Figure 1:**
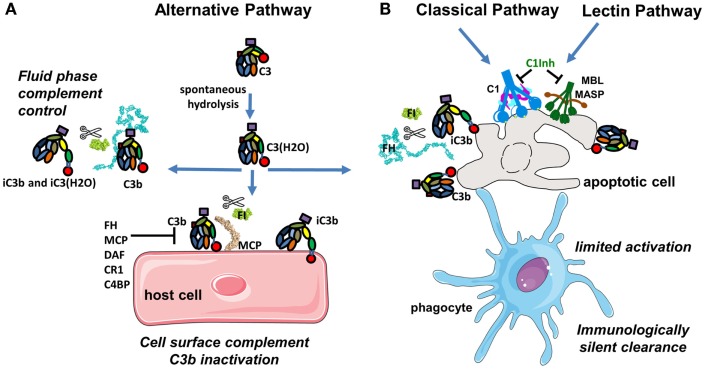
**Complement activation in physiological conditions**. **(A)** The alternative pathway is permanently active due to spontaneous transformation of bio-inactive molecule C3 to bioactive C3(H_2_O). This allows generation of C3b, which is rapidly inactivated by FH and FI in fluid phase or is covalently bound to the surface and then inactivated on host cells. **(B)** Classical and lectin pathway recognition molecules bind to apoptotic cells and together with C3b from the alternative pathway induce a low level of complement activation. Apoptotic cells are not lysed, but rapidly cleared by phagocytes in an immunologically silent manner. Host cells and plasma contain multiple regulatory proteins to assure that complement activation will be limited in physiological conditions.

**Figure 2:**
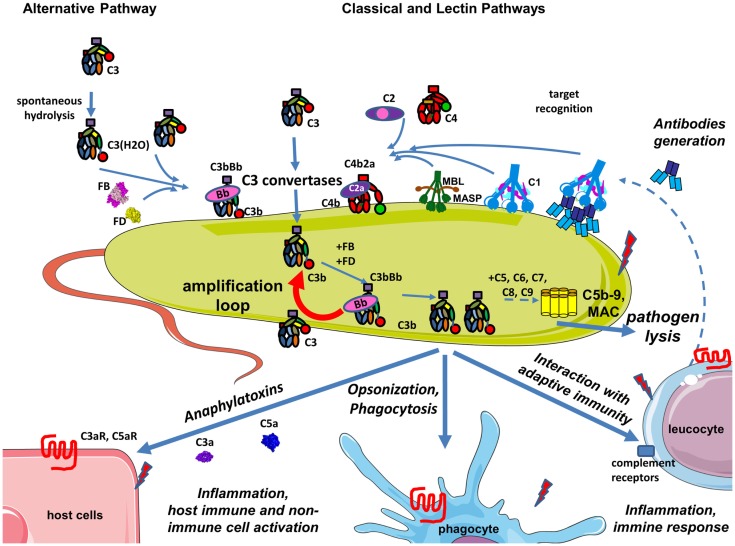
**Complement during infection with a pathogen**. The permanent activity of the alternative pathway allows it to immediately identify pathogens that are not specifically protected against complement. Danger-associated molecular patterns on its surface of pathogens are recognized by C1q, MBL, and ficolins allowing classical and lectin pathway activation, C3 convertase, C4b2a generation, and C3 cleavage. Opsonization due to covalent binding of C3b to the target is accelerated by the amplification loop of the complement pathways. The effector functions resulting from this complement activation are: pathogen lysis by C5b-9 membrane attack complex, opsonization and phagocytosis of the pathogen, activation of host immune and non-immune cells by complement anaphylatoxins, inflammation, stimulation of an adaptive immune response, and antibody generation. Secreted antibodies will bind to the pathogen and create immune complexes that will be recognized by C1q and will activate the classical pathway. Altogether these mechanisms contribute to pathogen elimination.

Complement is a central part of the innate immunity that serves as a first line of defense against foreign and altered host cells (1). The complement system is composed of plasma proteins produced mainly by the liver or membrane proteins expressed on cell surface. Complement operates in plasma, in tissues, or within cells (2). Complement proteins collaborate as a cascade to opsonize pathogens and induce a series of inflammatory responses helping immune cells to fight infection and maintain homeostasis. The complement system can be initiated depending on the context by three distinct pathways – classical (CP), lectin (LP), and alternative (AP), each leading to a common terminal pathway. In a healthy individual, the AP is permanently active at low levels to survey for presence of pathogens (Figure 1A). Healthy host cells are protected against complement attack and are resistant to persistent low-grade activation. The three pathways are activated on the surface of apoptotic cells, which are constantly generated within the body during normal cellular homeostasis (Figure 1B). This complement activation is tightly regulated to eliminate dying cells without further activation of other innate or adaptive immune components. Complement is only fully activated in cases of pathogen infection. During an infection, complement leads to inflammation, opsonization, phagocytosis, and destruction of the pathogen and ultimately results in activation of the adaptive immune response (Figure 2). Both inefficient and over stimulation of complement can be detrimental for the host and are associated with increased susceptibility to infections or non-infectious diseases, including autoimmunity, chronic inflammation, thrombotic microangiopathy, graft rejection, and cancer.

In this review, we discuss recent advances in the molecular and structural basis of activation and regulation of the complement pathways followed by the discussion of one complement-mediated disease – atypical hemolytic uremic syndrome (aHUS) to illustrate how the knowledge of the structure–function relationships between complement proteins helps to understand aHUS physiopathology and aid in the development of targeted therapy. In the second part of this review, published in the same issue of Frontiers in Immunology, we provide a detailed review of the literature related to the role of the complement system in immunity (3).

## Complement Activation during Normal Homeostasis and Pathogen Infection

The central component of the complement system is C3. The activation of each of the three pathways (CP, LP, and AP) results in cleavage of inactive C3 protein into the functional fragments C3a and C3b. C3a is an inflammation mediator and C3b is an opsonin, which can bind covalently and tag any surface in the immediate proximity to the site of its generation.

### Complement tick-over in the alternative pathway

In the plasma, during normal physiological conditions, the dominant active complement pathway is the AP (Figure 1A). The AP monitors for pathogen invasion by maintaining a low level of constitutive activation by a process known as tick-over (4). Tick-over is the spontaneous hydrolysis of a labile thioester bond, which converts C3 to a bioactive form C3(H_2_O) in the fluid phase (5). The rate of hydrolysis of C3 to C3(H_2_O) can be accelerated by interactions between C3 and a number of biological and artificial interfaces, including gas bubbles, biomaterial surfaces, and lipid surfaces and complexes (6). Upon hydrolysis, the thioester domain (TED) of C3 undergoes a dramatic structural change that exposes a binding site for another member of the AP called Factor B (FB). The C3(H_2_O)-bound FB is then cleaved by a serine protease (SP) Factor D (FD) allowing formation of a fluid phase C3 convertase complex C3(H_2_O)Bb. C3(H_2_O)Bb is able to interact and cleave native C3 molecules to C3a and C3b (5, 7–11). During normal physiological conditions, this C3 convertase constantly generates small amounts of C3b, which is able to bind covalently via its TED domain to any adjacent surface containing hydroxyl groups. Nevertheless, not all hydroxyl groups attract equally C3b (12). The -OH in the 6th position appears to be more reactive than the average -OH group in sugars. Therefore, the particular sugars composition of the pathogen surface will determine the efficacy of complement activation. C3b will bind covalently to a surface that is located within about 60 nm from the convertase, due to the fact that the half-life of the thioester in C3b is ≈60 μs with a poor attachment efficiency of 10% (13). On host cells, bound C3b molecules are rapidly inactivated by an army of membrane-expressed or fluid phase-recruited complement regulators (described in detail below). A tick-over mechanism for spontaneous activation of the CP has also been suggested, but the molecular interactions of this process are not well understood (14).

### Clearance of apoptotic cells

Apoptosis, programed cellular death, is a process of normal cellular homeostasis and in healthy individuals everyday billions of cells die by this mechanism. Complement activation occurs on apoptotic cells with low levels of C3b deposition to facilitate their elimination without releasing danger signals, which could lead to further immune responses (15, 16) (Figure 1B). This complement activation occurs by membrane alterations and by decreased expression of complement regulators on the membrane of apoptotic compared to resting cells. The silent clearance of the apoptotic cells is assured by the binding of the initiators of the CP (C1q) and LP [Mannose-Binding Lectin (MBL) and ficolins]. These initiator proteins interact with receptors on phagocytic cells (immature dendritic cells or macrophages), which elicit immune tolerance and prevent immune responses toward self-antigens (17–20).

### Pathogen elimination

On pathogens that lack specific regulators of complement, C3b interacts with FB and FD to form a surface-bound C3 convertase as part of the AP, which cleaves C3 into C3a and C3b. Maximum complement activation is achieved during pathogen recognition leading to a pro-inflammatory milieu, contributing to generation of an adaptive immune response and rapid elimination of the pathogen (Figure 2). Complement-derived anaphylatoxins have potent inflammatory mechanisms including recruitment of phagocytes to the site of infection and activation of leukocytes, endothelial cells, or platelets. Upon activation, terminal complement components also have direct lytic capacity to kill pathogens.

The CP and LP have a critical role in pathogen recognition and initiation of the complement cascade. However, the AP assures more than 80% of the terminal complement activity during pathogen recognition (21). Additional AP C3 convertases are formed on the C3b molecules generated either by CP activation or the AP C3 convertases. This chain reaction amplifies opsonization of the target and increases generation of anaphylatoxins. This amplification loop augments the effect of all pathways and is the heart of the complement cascade (22).

## Structural Basis of Complement Activation and Regulation

### Target recognition and initiation of complement pathways

The CP and LP have clearly identified recognition molecules, C1q, MBL, and ficolins, which trigger each pathway only when and where it is necessary. The recognition event induces a structural change in the recognition molecule, which in turn induces the activation of enzymes able to cleave the subsequent molecules in the cascade and generate the central enzymatic complexes of complement, CP and AP C3 convertases. The AP lacks a traditional target recognition molecule as an initiator. However, several molecules, such as properdin and P-selectin, can recruit C3(H_2_O) and C3b to the cell surface and serve as local initiators of the AP.

### Recognition molecules of the classical and lectin complement pathways

**Figure 3:**
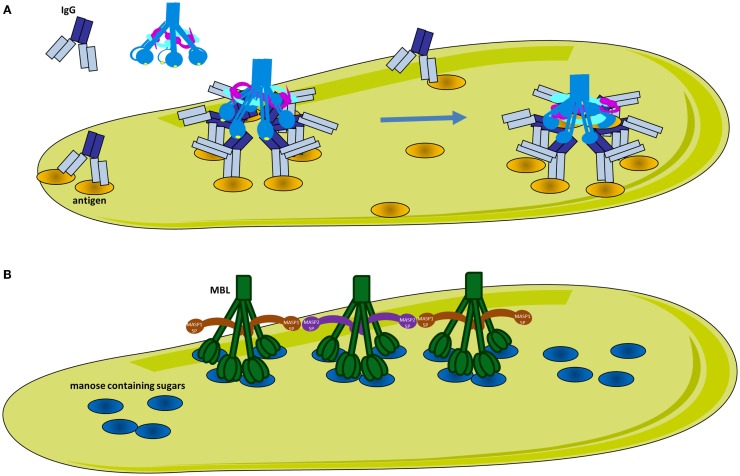
**Classical and lectin pathway activation**. **(A)** Activation of the classical pathway. Multiple adjacent IgG molecules are needed to bind C1q. IgG interacts with its target antigen forming specific circular structures. A single FAB binds to the antigen, while the other does not. The movement of the Fc domain exposes the C1q-binding sites allowing complementarity with the six globular domains of C1q (gC1q). The number of engaged IgG molecules will determine the compatibility of the immune complex with C1q and hence the strength of classical pathway activation. C1q circulates in plasma-associated with the serine proteases C1r and C1s, forming inactive C1 complex. After binding, the target C1q undergoes a conformational change to increase the angle between its collagenous stalks (CLR). This conformational change activates C1r, which in turn activates C1s. **(B)** Activation of the lectin pathway. MBL recognizes mannose containing sugars on pathogens. MBL circulates associated with serine proteases MASP-1 or MASP-2. Upon target binding, juxtaposition of MASP-2 and MASP-1 containing MBL complexes is required for MASP-1 to activate MASP-2.

Complement pathway and LP are triggered after interaction of a pattern-recognition molecule with the target structure. The recognition molecule of the CP, C1q, has an extra-hepatic origin and is produced mainly by immature dendritic cells, monocytes, and macrophages (23). It has a complex, described as a “bouquet of flowers” topology (Figure 3A), composed of 18 chains of three types (A, B, and C), forming six globular target recognition domains (gC1q) attached to a collagen-like region (CLR). Each gC1q domain carries a Ca^2+^ ion, which maintains domain stability and regulates the electrostatic field (24). C1q recognizes mostly charged patterns and can bind to more than 100 different target molecules, including IgG and IgM containing immune complexes and surface-bound pentraxins [C-reactive protein (CRP), pentraxin 3 (PTX-3)] (25). Therefore, CP can be activated in either an immune complex-dependent and -independent manner. Mapping of the target molecule-binding sites on gC1q by a site-directed mutagenesis, revealed that charged residues on the apex of the gC1q heterotrimer (with participation of all three chains), as well as, the side of the B-chain are crucial for binding to IgG, IgM, CRP, and PTX-3. These binding sites are not identical, but partially overlapping (24, 26–29). C1q recognizes pathogen-associated molecular patterns including lipopolysaccharide (LPS) (30) and bacterial porins (31). The gC1q domain also recognizes molecules, exposed on the surface of dying cells (32, 33), including phosphatidylserine (34, 35), double stranded DNA (36, 37), glyceraldehyde-3-phosphate dehydrogenase (GAPDH) (38), annexins A2 and A5 (39), and calreticulin (35, 40–42). The most well-characterized target recognition molecule of the LP is the MBL, which recognizes carbohydrates (43). MBL has a similar overall structure to C1q, but exists in multiple oligomeric forms (trimers, tetramers, and higher ordered oligomers) (Figure 3B). C1q and MBL associate in a Ca-dependent manner with SP complexes, consisting of C1r and C1s for the CP (44, 45) and MBL-associated serine proteases (MASP) for the LP (46, 47). In absence of Ca^2+^ ions (such as in plasma samples collected in EDTA), C1q and MBL cannot interact with C1r_2_C1s_2_ and MASPs, respectively and CP and LP activation is prevented. In the presence of Ca^2+^ ions, after activation, SPs cleave subsequent complement components C4 and C2. The resulting complex C4b2a is the C3 convertase for CP and LP. This C3 convertase has enzymatic activity and is able to cleave the central complement component C3 to bioactive fragments C3a and C3b.

#### Mechanism of Activation of the Classical Pathway

Recent studies have shed light on the molecular mechanisms of activation of CP and LP. It has long been established that C1q requires one surface-bound IgM or several IgG molecules in close proximity in order to interact with several of its globular domains and to activate complement. However, the molecular mechanisms and the C1-antibody stoichiometry required for optimal activation remain poorly understood (48). IgM is a planar polymeric molecule (pentamer or hexamer), in which C1q-binding sites are hidden. A conformational change occurs upon binding to an antigen (staple conformation), leading to exposure of C1q-binding sites. Contrary to IgM, IgG is a monomer and despite the presence of the C1q-binding sites, only very low affinity binding can be achieved. The epitope distribution of the antigen and the density of the IgG binding determine the level of complement activation, however the molecular mechanisms were unknown until recently. Diebolder and colleagues demonstrated that specific non-covalent interactions between Fc fragments of IgG and formation of ordered antibody hexamers on the antigen surface are needed for efficient C1q-binding (Figure 3A) (49). Their proposed model could explain the strong antigen and epitope dependency of complement activation. Efficient C1q-binding could only occur upon formation of a platform of IgG Fc fragments with a steric compatibility for gC1q domains. Clustering of IgG molecules on the antigen surface could be affected by antigen size, density, and fluidity (50, 51) such that smaller antigen-antibody complexes will allow only moderate complement activation. In addition, binding stoichiometry is further complicated by antibodies fluidity on surfaces of regularly spaced epitopes. It has been demonstrated that IgG exhibit “bipedal” stochastic walking forming transient clusters that might serve as docking sites for the C1q-binding and complement activation (52).

**Figure 4:**
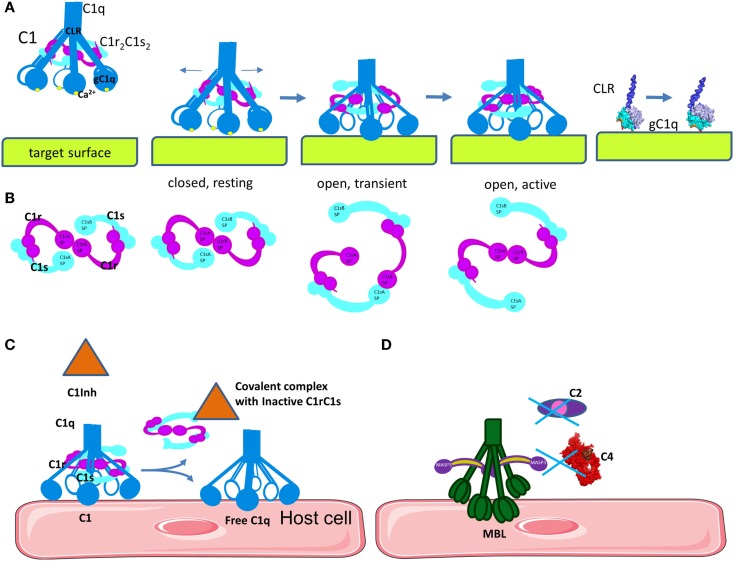
**Mechanism of C1 classical pathway activation and regulation**. **(A)** Structural changes in C1q are necessary to induce auto-activation of C1r and activation of C1s. Upon binding of the inactive, closed C1 complex, electrostatic interactions with a target surface may alter the electrostatic field of the domain. This will induce a rotation of gC1q, leading to opening of the angle between the CLRs. A part of the binding site on gC1q apex will be lost, but new links will be formed with the side surface of the B-chain. **(B)** Concomitant with the structural changes in C1q, C1r_2_C1s_2_ complex will pass from closed, inactive eight-shaped conformation to an active, S-shaped conformation, allowing C1r auto-activation and further C1s activation by C1r. **(C)** The C1 inhibitor is a serpin that binds covalently to the active site of C1r and C1s, blocking their function. It also dissociates C1r_2_C1s_2_ from C1, releasing free C1q. C1 inhibitor also inhibits the lectin pathway by binding to MASP-1 and MASP-2. **(D)** MBL can bind to MASP-3, MAp44, or MAp19, which cannot cleave C4 and C2.

Once C1q binds to its target surface, a conformational change is required to transmit the signal from the gC1q domain via the CLR to induce auto-activation of C1r (Figure 4A) (53). Molecular modeling, mutagenesis, and disease-associated mutation analysis revealed the structure of the C1r_2_C1s_2_ binding site in the cone of collagenous arms of C1q (44, 54, 55). The C1r_2_C1s_2_ proenzyme tetramer within the C1 complex in a resting state adopts an eight-shaped form (Figure 4B). Upon activation, conformational changes in the tetramer allow transition to an S-shaped active form, passing through a transition state. This conformational change allows auto-activation of the C1r SP domain. Subsequently, activated C1r will cleave and activate C1s. The driving force for this auto-activation of C1r is an increase of the angle between the collagen stalks of C1q (48). However, the mechanism for this structural change is poorly understood. Mutagenesis experiments have revealed that residues on the apex and the lateral surface of the B-chain of gC1q are important for IgG, IgM, CRP, or PTX-3 interactions (27–29). Taking into account the surface morphology of gC1q and its targets, it is difficult to contemplate how these residues can engage simultaneously in binding. One possibility is that these residues form binding sites, which make contact with the target subsequently and not simultaneously (Figure 4A). These data, combined with the importance of the Ca^2+^ ions for the electrostatic field of C1q (24), and the induced conformational change leading to an increased angle between the collagenous stalks by gC1q and target interaction (48, 56–60) has led to the proposal of an electrostatic model for the activation of the C1 complex (Figure 4A). This model suggests that the increase in the angle between the collagen stalks occurs because of a rotation of the gC1q domains, driven by electrostatic interactions between gC1q and the target molecule (IgG, IgM, or CRP) (24). Interactions between the negatively charged binding sites on the target may cause a removal of the Ca^2+^ ion from gC1q. Loss of Ca^2+^ changes dramatically the size and orientation of the electric moment vector of gC1q. This electrostatic change can induce the rotation of gC1q allowing it to engage the lateral surface of the B-chain. This rotation may provide the mechanical stress necessary for the transition of the C1r_2_C1s_2_ complex from closed, inactive eight-shaped conformation to an active, S-shaped conformation, allowing C1r auto-stimulation and further C1s activation by C1r. In this active form allows the tetramer to unfold and to extend its C1s ends outside the C1q cone for interaction with C4 and C2. Cleave of C4 and C2 by C1s allows formation of the CP C3 convertase in the immediate proximity to the C1 complex-binding site (44, 45, 53, 61). It is a matter of debate whether the C1s catalytic domain faces the exterior or the interior of the C1q cone. If the recognition and cleavage of C4 occurs inside the cage-like structure of the cone of C1 (62), this may increase the efficacy of the covalent binding of the bioactive cleavage product C4b to the surface. However, it is still unclear what would be the driving force to assure the entrance of both C4 and C2, as well as the substrate molecules of C3, in this confined space. These data imply that one C1 complex will have limited efficacy, generating only one or two C3 convertases as a result of steric hindrance. It is too few compared to the experimental evidence that one activated C1 complex generates about 35 C4 molecules during its lifespan (63). In order to understand the exact architecture of the C1 complex, further investigation will be required.

#### Mechanism of Activation of the Lectin Pathway

The pattern-recognition molecules of the LP are MBL, collectins, as well as ficolins H, L, and M (64, 65). The LP pattern-recognition molecules have an N-terminal collagenous region similar to C1q, however their C-terminal domains differ from gC1q. Collectins contain carbohydrate recognition domains, which recognize sugar patterns. MBL, which belongs to the collectin family, recognizes terminal monosaccharide exposing horizontal 3′- and 4′-OH groups (glucose, mannose, and *N*-acetyl-glucosamine) in a Ca-dependent manner. These sugars are rarely present on host proteins and cell surfaces, but frequently expressed on bacteria, viruses, and dying cells. Ficolins are associated with MASPs protein in the circulation and have C-terminal recognition fibrinogen-like domains, which are able to bind acetyl groups, such as *N*-acetyl-glucosamine, on the surface of bacteria. Following binding, MASPs associated with MBL or ficolins are activated and result in the cleavage of C4 and C2 (66, 67). Similar to C1q, stable binding can be achieved only when the ligands are clustered on the surface forming a specific pattern. This complex can simultaneously engage several carbohydrate recognition domains or fibrinogen-like domains for collectins and ficolins respectively.

Despite the similarity between the architecture of the C1 and MBL/MASP complexes, the mechanism of activation of the LP is different than the classical one (64). While in the CP each C1 complex carries both C1r and C1s, the majority (~70%) of the MBL molecules present in plasma are associated with only one homodimer of either MASP-1 or MASP-2 to assure their separation (Figure 3B) (68). In physiological conditions, MASP-1 is required for the activation of MASP-2 and both activated proteases can cleave C2 while MASP-2 can also cleave C4. Auto-activation properties confer to MASP-1 a fluctuating state between inactive and active-like conformations, giving it a key role in LP activation (69–74). Auto-activation of MASP-2 provides a residual capacity (~10%) to cleave its natural substrate C4 in zymogen form (75). Since MASP-1 and 2 are associated with different MBL or ficolin molecules, they are required to juxtapose their recognition molecules on ligand surfaces to facilitate activation of different MASPs (76). Therefore, MASP-1 from one complex will activate MASP-2 from the adjacent complex, allowing C4 cleavage (77). MASP-3 also influences LP activation.

The described mechanisms of activation of the CP and LP illustrate two of the key characteristics of the complement cascade. Complement activation relies on the versatility of the target patterns recognition molecules (C1q, MBL, ficolins) that can discriminate between self and non-self and bind to pathogen- or danger-associated molecules. These molecular patterns are often generated after a specific conformational changes, such as with IgM or particular clustering, such as with IgG, CRP, or pathogen-associated carbohydrates. Complement is driven by these conformational changes that transmit a signal as a result of the recognition event to the subsequent complement components, activating them or modulating their function.

#### Regulation of the Classical and Lectin Pathways Initiation

Activation of the CP is controlled by a serpin molecule, C1 Inhibitor (C1Inh). C1Inh binds and inactivates C1r and C1s, leading to dissociation of the C1 complex and liberation of free C1q leaving an inactive covalent complexes between C1Inh and C1r or C1s (Figure 4C) (78, 79). C1Inh is thought to bind to and stabilize unactivated C1r and C1s in the C1 complex thus retarding their spontaneous activation (80), but this function is poorly studied. C1Inh has additional functions outside complement inactivation, related to kinin pathway (a plasma system related to inflammation, vasodilatation, and pain). Angioedema is disease caused by hereditary or acquired C1Inh deficiency. The edemas are triggered by increased permeability of the blood vessels in response to elevated levels of bradykinin as a result of the C1Inh deficiency. Recombinant and plasma-derived C1Inh are approved therapeutic agents for hereditary angioedema (81).

C1q inhibitors released under physiological or pathological conditions such as chondroitin-4 sulfate proteoglycan and the hemolysis derivative heme can bind to C1q and inhibit the CP (55, 82). The mechanism of action of heme, as well as a number of synthetic C1q inhibitors, rely on binding to gC1q and alteration of its electrostatic properties (55, 83). Calreticulin, released during cell death or from parasites can also act as an inhibitor of C1q to prevent CP activation (84, 85).

Inhibition of the LP is influenced by MASP-3, MAp44, and MAp19 proteins, which share high sequence homology with MASP-1 and -2 and have similar binding affinity to MBL and ficolins (70, 86). These proteins may compete with MASP-1 and -2, but are unable to cleave MASP, C2, and C4 preventing further activation of the LP cascade (Figure 4D). In addition, C1Inh is able to control LP activation by inhibiting MASP-1 and MASP-2 but not MASP-3 activity (87, 88).

## Platforms for Surface Assembly of the Alternative Pathway C3 Convertase

**Figure 5:**
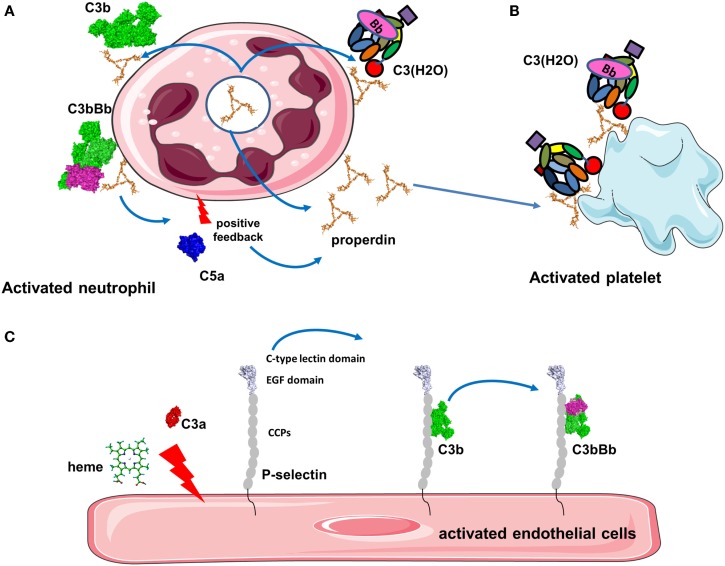
**Platforms for complement activation**. **(A)** Properdin is released from activated neutrophils and is bound to the cell membrane where it recruits C3b to form the alternative pathway C3 convertases. C5a then activates additionally neutrophils and they secrete more properdin. This installs a vicious cycle of neutrophil and complement activation. **(B)** Properdin released from neutrophils or in the plasma binds to activated platelets promoting C3(H_2_O) recruitment and complement activation. **(C)** Stimulation of endothelial cells with C3a, heme, or other agonists induces expression of P-selectin. P-selectin contains CCP domains and binds C3b, promoting formation of C3 convertases that generate more C3a to stimulate cells.

C3b can bind to the cell surface not only via its own thioester bond, but also by interacting with surface molecules that serve as platforms for C3b recruitment (Figure 5). Recently, it has been observed that C3b and C3(H_2_O) can also bind to the cell surface by these platforms resulting in local activation of the AP. Although activated platelets are the predominant cell type that binds C3(H_2_O) (89, 90), the exact molecules it binds are not yet well defined. Nevertheless, several molecules are likely candidates for activated C3 recruitment on different cell types.

Properdin, which stabilizes the alternative C3 convertase (91), is able to bind pathogens, activated or damaged host cells to induce stimulation of the AP (Figure 5A) (92, 93). Properdin recognizes heparin and heparan sulfate on tubular cells leading to complement activation (94). Properdin contributes to the AP activation on human neutrophils and has been observed in neutrophil-mediated diseases (95). Degranulation stimuli on neutrophils induce low properdin release and deposition, triggering AP by recruiting C3b and promoting C3 convertase formation. Plasma properdin is also required for an efficient C3b feedback loop and amplified AP activation (95). Generation of C5a also further stimulates properdin release and amplifies complement activation and neutrophil stimulation. Moreover, it has been demonstrated that myeloperoxidase secreted by neutrophils during degranulation binds to properdin and leads to the AP activation (96). Properdin released by activated neutrophils can also bind to activated platelets. In the absence of C3, properdin can also bind directly to platelets after interaction with a strong agonist and serves as a platform for recruitment of C3b or C3(H_2_O) and C3 convertase formation (Figure 5B) (90). This complement activation may contribute to localization of inflammation at sites of vascular injury and thrombosis.

Another protein, which can recruit C3b to a cell surface is complement Factor H (FH)-related protein 4A (CFHR4A). This protein shares sequence homology with FH and is able to bind C3b via its C-terminal domain. CFHR4A lacks regulatory domains and cannot inactivate C3b. Even more, it has been suggested that CFHR1A can serve as a platform for the alternative C3 convertase formation. Formed convertase on CFHR4A had a higher resistance to FH-mediated decay.

P-selectin (CD62P) recruits leukocytes via binding to P-selectin glycoprotein ligand 1 (PSGL-1) (97) and has been described to bind C3b on the cell surface leading to the activation of AP (Figure 5C) (98, 99). Morigi et al. showed the effect of P-selectin as a platform for C3 convertase formation *in vitro* and in a murine model of Shiga toxin (Stx2)/LPS-induced hemolytic uremic syndrome (HUS) (99). P-selectin expression was partially triggered by the anaphylatoxin C3a contributing to a vicious circle of complement activation aggravating microvascular thrombosis HUS pathology (99).

Another activator of C3 convertase, heme, is released from hemoglobin during hemolysis, where it stimulates the AP. Heme induces deposition of C3 activation product in erythrocytes and has been shown to play a role in malaria pathogenesis (100, 101). Heme binds C3 (not C3b), likely near to the TED domain, leading to the generation of C3(H_2_O) and homophilic C3 complexes associated with overactive C3/C5 convertases (102). Furthermore, *in vitro* experiments on human EC have shown that heme-induced mobilization of specific EC granules that store von Willebrand Factor and P-selectin called Weibel Palade bodies, is at least in part induced by TLR4 (102, 103). This TLR4 stimulation lead to degranulation of P-selectin accompanied by C3b and C3(H_2_O) binding to the cell surface of EC. Heme is a hydrophobic molecule that binds to lipid bilayers and it is hypothesized that cell-bound heme may serve as a platform to recruit C3(H_2_O) (102).

Collectively, these examples lead us to propose a general mechanism for a positive feedback loop implicating protein platforms in tissue damage. An initial trigger will stimulate the cell to either express a platform protein (properdin for neutrophils or P-selectin for EC and platelets) or to bind molecules from the fluid phase (properdin, CFHR4A, or heme in case of hemolysis). The type of the platform will likely depend on the cell type, location of activation, and other yet undiscovered factors. C3(H_2_O) will bind to these platforms and will initiate local complement activation and C3b deposition. The amplification loop will generate C3a and C5a, which upon binding to their receptors (described below) will augment cell activation and increase expression of platform proteins stored in intracellular granules or recruited from the plasma. These events will form an intensified circle resulting in local inflammation, thrombosis, and tissue damage.

## Structure and Function of the C3 Convertases

### Alternative pathway C3 convertase

**Figure 6:**
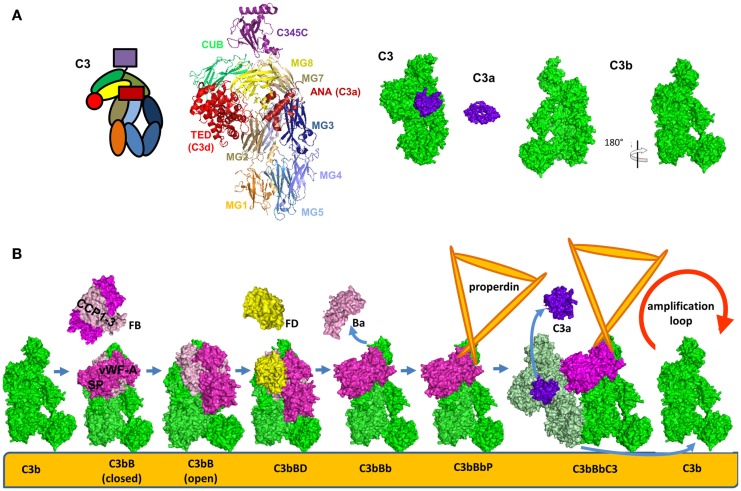
**Alternative pathway C3 convertase**. **(A)** Structure and domain organization of the central complement component C3 and its cleavage fragments C3b and C3a. C3b is shown in two orientations to illustrate the surfaces containing the ANA domain and the opposite surface, carrying FB and FH binding sites. **(B)** Steps of formation of the alternative pathway C3 convertase. C3b is shown in green, FB in magenta, FD in yellow, C3a is in violet, and the substrate molecule C3 – in light green. For these molecules, the available crystal structures were used for the visualization. The C3bBbC3 complex is visualized based on molecular modeling. Properdin, for which a crystal structure is not available, is depicted in orange.

The structure and function of the AP C3 convertase has been dissected during the few last years. Upon cleavage and removal of C3a, C3b undergoes a dramatic structural change (Figure 6A) leading to exposure of novel binding sites. This allows recruitment of FB which binds in a Mg^2+^-dependent manner and yields the pro-convertase C3bB (Figure 6B) (104). This interaction occurs via the Von Willebrand Factor A-type (VWF-A) domain and three complement control protein (CCP1-3) domains of FB (104, 105). The catalytic SP domain of FB undergoes large conformational changes oscillating between a closed (loading) and an open (activation) forms (Figure 6B) (104–106). In the open (activation) conformation, the scissile bond is exposed and the FD binding site is formed correctly.

Factor D is synthesized in an inactive pro-FD enzyme lacking proteolytic activity (107). It was suggested that this zymogen form can be cleaved by MASP-1/3 into a form with limited activation to support the basal levels found in the AP (108, 109) and becomes fully activated only upon binding to C3bB open complex. The physiological relevance of MASPs-mediated cleavage of pro-FD is still being debated. MASPs cleavage is not the only mechanism for FD activation, since mice deficient in MASP-1/3 have reduced but detectable AP activity (110) and the only patient found to be deficient in MASP-1 and -3 was reported to have a normal AP activity (111). Further studies are needed to elucidate the mechanism of FD activation in mice and men. Insights into this pathway could help lead to the development of MASP-1 inhibitors as a strategy to treat renal diseases associated with uncontrolled AP activation.

Upon activation, FD binds to and cleaves C3b-bound FB releasing the N-terminal fragment Ba (comprising the CCP1-3 and the αL helix). This results in the formation of the C3 convertase of the AP C3bBb (Figure 6B) (112). The Bb fragment consists of a VWF-A and a SP domain. The SP domain undergoes a new structural change and is positioned in a conformation, similar to the closed form of the pro-convertase (Figure 6B) (113). A substrate molecule of C3 binds then to the alternative C3 convertase and is cleaved generating new C3b and C3a molecules (Figure 6B) (113).

Factor B binds in a similar manner to C3(H_2_O) to form the alternative C3 convertase [C3(H_2_O)Bb] in the fluid phase (114). Even if FB has a higher affinity to C3(H_2_O) than to C3b, the convertase activity remains lower than the C3bBb complex, as measured by C3a released. The fluid phase convertase C3(H_2_O)Bb also has higher resistance to decay by complement inhibitors (114).

### Classical pathway C3 convertase

**Figure 7:**
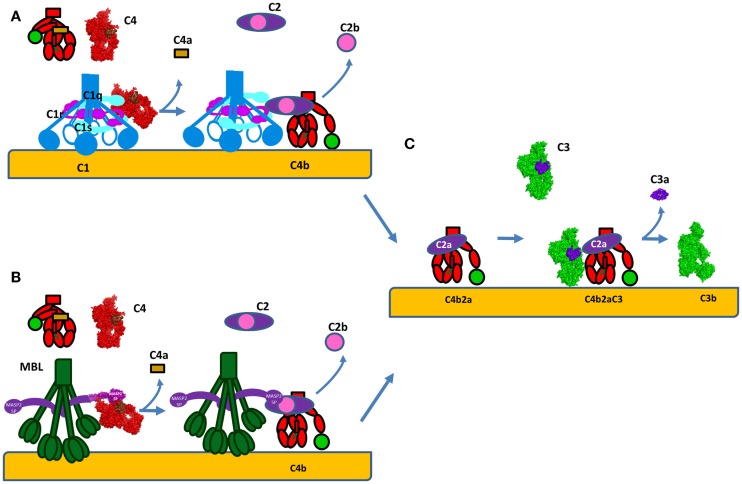
**Formation of the C3 convertase by the classical and lectin pathways**. **(A)** C1s or **(B)** MASP-2 will cleave C4 into bioactive fragment C4b that bind covalently to the surface of cells and interacts with C2. The small fragment C4a is released. Following, the same enzyme will cleave C2 to generate the classical pathway C3 convertase C4b2a. **(C)** C4b2a will interact with C3 cleaving it and releasing the bioactive fragments C3a and C3b. C3b will bind covalently to the surface and allow formation of alternative pathway C3 convertases C3bBb via the amplification loop. The C3a generated is a pro-inflammatory anaphylatoxin. The C4 molecule is presented in red, with brow colored the ANA domain, which will become C4a after cleavage and in green – the TED domain, which will become C4d after cleavage. The crystal strictures of C4, C4b, and C2a were used for the representation. The CP C3 convertase C4b2a is modeled based on the structure of the AP convertase C3bBb, with which it shares high homology.

The structure and mode of action of the CP C3 convertase is not well understood. C4 and C2 share high degree of sequence and structure homology to C3 and FB respectively, thus the mechanism of formation of the classical C3 convertase may be similar to the well-studied AP C3 convertase, described above (Figure 6). C4 is cleaved by activated C1s or MASP-2 to bioactive fragment C4b and a small fragment C4a (Figures 7A,B). C4b contains an internal thioester bond, similar to that in C3b, and forms covalent amide or ester linkages with the antigen–antibody complex or the adjacent surface of a cell. C4b binds C2 in Mg^2+^-dependent manner. C2 is then cleaved by C1s or MASPs. Since the concentration of C2 is about 20–30-fold lower compared to C4, one active C1 complex can cleave about 35 C4 molecules, while only 4 C2 will be cleaved for the same time (63). The larger fragment C2a remains bound to C4b and forms the CP C3 convertase C4b2a (Figure 7C) (115) and the smaller fragment C2b is released in the circulation. Historically, C2b was considered to have kinin-like properties, but recent reports failed to confirm this function (116, 117).

The C3 convertases are an excellent example of a general mechanism that governs different steps of the complement cascade. Each subsequent step can only occur after a conformational change, triggered by the preceding step, thus assuring the temporal and specific control of this powerful destruction cascade (118).

### Stabilization of the alternative pathway C3 convertase

The C3bBb is a short-lived complex with a half-life of about 90 s (119) and, therefore a stabilization of this complex is required to assure efficient host defense (Figure 6B) (93). The AP C3 convertase is stabilized 5- to 10-fold by association with properdin (91). Properdin (120) is secreted by monocytes/macrophages and T lymphocytes (121, 122) and is stored in secondary granules of neutrophils (95, 123) and mast cells (124). Properdin is composed of identical rod-like protein subunits (125) that are associated head-to-tail to form cyclic dimers, trimers, and tetramers that resemble rods, triangles, and squares, respectively. The function of properdin is dependent on its level of polymerization, with the tetramer being approximately 10-fold more active than the dimer (126). Purified properdin results in aggregates and has artificially high binding activity (127, 128). Properdin binds C3bBb, as well as, the pro-convertase C3bB and C3b (92). Properdin interacts both with the C345C domain of C3b and the VWF-A domain of Bb, in order to stabilize the convertase (129). Interestingly, electron microscopy studies visualized that properdin binding induces a large displacement of the TED and CUB domain of C3b (129). These structural changes distort the FH binding site (130–132), which may explain the relative resistance of the stabilized C3 convertase to decay by FH.

Recent studies of Hourcade and colleagues demonstrated that properdin is not merely a stabilizer of the C3 convertase, but also a pattern-recognition molecule that binds to microbial surfaces including glycosaminoglycans (GAGs), apoptotic, and necrotic cells providing a platform for C3 convertases assembly (93).

## Negative Regulation of the C3 Convertases

The amplification loop is the balance between two competing cycles, acting on the C3b–C3 convertase formation, which enhances both amplification and downregulation via the C3 breakdown cycle. Complement amplification depends on the balance between binding rates of each reaction (22). To regulate activation, several inhibitors of complement pathways, primarily against AP, exist in the fluid phase and on host cells. Complement inhibitors have overlapping functionality. FH works as a soluble inhibitor of the alternative C3 convertase, while membrane cofactor protein (MCP), decay acceleration factor (DAF), and complement receptor 1 (CR1) work as membrane inhibitors. FH is a specific cofactor for C3b and C4 binding protein (C4BP) primarily is a cofactor for C4b, while MCP and CR1 act as cofactors for the inactivation of both C3b and C4b via Factor I (FI).

### Inactivation of C3b and C4b by factor I

Factor I is a SP found in the plasma that cleaves C3b in presence of different cofactor molecules, such as FH, MCP, CR1, or C4BP (Figure 9A). The protease activity of FI leads to the generation of degradation product of C3b, iC3b, which is unable to bind FB (133). A zymogen form of FI has not been detected in the circulation. In fact, FI circulates in a proteolytic form but in inhibited conformation. The activity of the light chain of FI is allosterically inhibited in the circulation by a non-catalytic heavy-chain (134). In presence of its cofactors, the non-catalytic heavy-chain of FI is released and it is able to cleave C3b between Arg1281 and Ser1282 to give the iC3b fragment (134, 135). Molecular modeling suggested that FI binds FH1–4 and C3b simultaneously in a groove between two proteins, which is in agreement with the hypothesis that FI binds CCP1–3 of FH and C345C of C3b (132).

#### Factor I Cofactors

**Figure 8:**
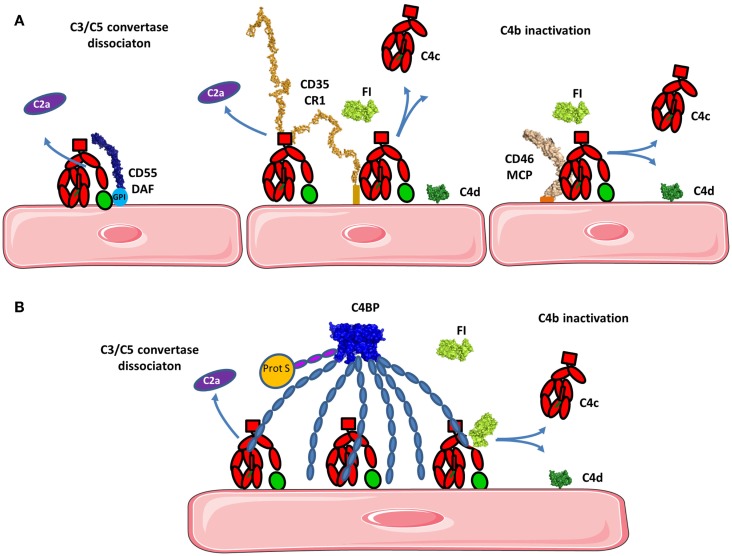
**Regulation of the classical and lectin pathways C3 convertase**. To avoid overactivation, the CP and LP tightly regulate signaling. **(A)** If a C4b2a C3 convertase is formed, it will be rapidly dissociated by DAF and/or CR1 depending on the cell type. Bound C4b will be inactivated by FI in presence of cofactors such as CR1 and/or MCP. C4d will remain bound to the surface and C4c will be released. **(B)** C4BP can act in fluid phase as well as on the cell surface. C4BP has an octopus structure and interacts with several C4b molecules. It dissociates the C3 convertase and serves as a cofactor for FI in the cleavage of C4b to inactive fragments C4c and C4d. The structures of the complexes of C4b with the regulatory proteins have not been resolved yet, therefore the proteins are depicted in proximity one to another, represented by their known structures, but no complex could be reliably modeled.

**Figure 9:**
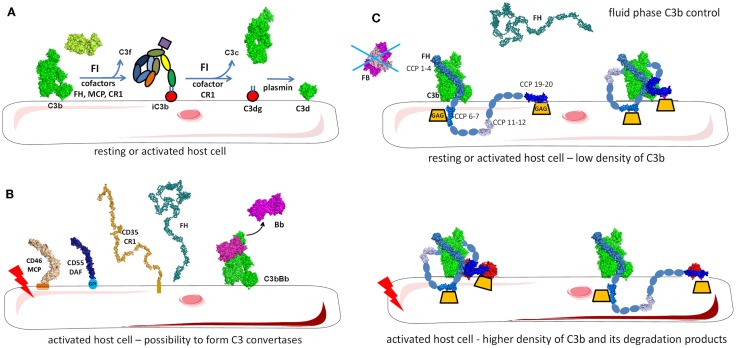
**Regulation of the alternative pathway**. **(A)** FH as a master regulator of C3b in the fluid phase and on the cell surface. FH binds to C3b in fluid phase preventing novel convertase formation. FH may bind to C3b and GAGs on the cell surface and the architecture of the complex depends on the level of activation of the cell and the density of deposited C3 fragments. Resting cells have only a few C3b molecules that are deposited and FH binds to them with the regulatory domains CCP1-4. CCP7 and CCP20 interact with GAG on the membrane. Alternately, CCP19 may bind to the TED domain of C3b allowing CCP20 to interact with GAGs. If the cell is activated and C3b and C3d (or two C3b molecules) are deposited in close proximity, FH may bind to two of these molecules, allowing GAG binding by CCP20. **(B)** On resting cells, C3b will immediately be inactivated to iC3b by the action of FI and the assistance of cofactors (FH, MCP, CR1). iC3b cannot bind FB and forms C3 convertases. Only the cofactor CR1 allows FI to execute a second cleavage generating C3c (released in the fluid phase) and C3dg, which remains bound to the cell. C3dg is rapidly transformed to C3d by tissue proteases. **(C)** If the host cell is activated, the complement control will not be sufficient to prevent any complement deposition and C3 convertases could be formed. To avoid cell damage, these convertases need to be dissociated. Multiple complement regulators such as DAF, CR1, and FH decay the C3bBb complex formed on host cells. Remaining C3b will be inactivated by FI, using FH, MCP, or CR1.

Membrane cofactor protein, DAF, and CR1 (CD35) serve as cofactors for FI-mediated proteolysis of C4b and C3b. MCP is composed of 4 extracellular CCP domains expressed on all nucleated cells (136) and CR1 contains 30 extracellular CCP domains and is expressed on leukocytes, erythrocytes, and glomerular podocytes. MCP N-glycosylation on CCP2 and CCP4 is essential for MCP inhibitory activity (137). CCP1–4 of MCP binds to C3b and C4b and is structurally similar to the four N-terminal domains of FH (described below), both proteins serve as cofactors for FI for C3b inactivation (Figure 9A) (132, 138–140). FH only induces C3b-degradation and is inefficient for C4b. CCP-3 and -4 of MCP are responsible for binding to C3b and C4b, while CCP1–2 only interacts with C4b (Figure 8A) (141). The binding site for MCP on C3b is partially overlapping with the site for CCP-3–4 on FH (142).

The first 28 CCP of CR1 are 4 long homologous repeats (LHR) for 7 CCP domains containing the binding sites for C3b and C4b (143, 144). C3b and C4b-binding sites located in CCP8–10 (LHR2) and CCP15–17 (LHR3), respectively, are responsible for FI cofactor activity (143, 145). CCP15–17 has a major role in C3b/C4b inactivation (146). CCP15 carries a positively charged region essential for the C4b-binding and a basic region in the CCP16 that is necessary for C3b-binding. Although the architecture of the CR1/C3b and CR1/C4b is not well defined, it is known that CR1 interacts with the α′NT region of C3b (residues 727–767), which overlaps with the FH CCP1-binding site, but not MCP-binding site (132, 133, 142, 147).

C4b can be inactivated by the action of C4BP, a plasmatic cofactor for FI (Figure 8B) (148–151). C4BP has a complex, octopus-like structure comprised of CCP domains containing α- and β-chains (152, 153). The first three CCP domains of each α-chain are involved in cofactor and convertase dissociation functions. A maximum of four C4b molecules can simultaneously interact with the α-chains of one C4BP molecule (154). The β-chain binds coagulation protein S and contributes to regulation of the coagulation cascade (155).

Complement receptor 1 is a unique cofactor of FI due to its ability to induce further cleavage of iC3b, generating C3c and C3dg degradation fragments (Figure 9A) (133), where FH, MCP, and C4BP only induce the cleavage of C3b to iC3b.

Several proteins have been shown to enhance FI-mediated cleavage in the presence of cofactors. Thrombomodulin binds to C3b and FH and negatively regulates complement by accelerating FI-mediated inactivation of C3b in the presence of FH and C4BP (156). Von Willebrand factor also enhances the efficacy of FH as a cofactor for FI (157). In specific *in vitro* buffer conditions, Von Willebrand factor has been suggested to have direct cofactor activity, but the physiological function of this interaction requires further validation (158).

### C3 convertase dissociation

The AP C3 convertase is dissociated and Bb is released primarily by the action of CCP1–4 in FH (described below). CR1 dissociates the C3 and C5 convertases. CCP1–3 of CR1 carries a binding site for C4b allowing it to induce accelerated decay of the C3 convertase in the CP and AP (Figures 8A and 9B) (143, 159). C4BP prevents the formation of the classical C3 and C5 convertases (148–151).

Decay acceleration factor accelerates decay of the C3 and C5 convertases in the CP and AP (Figures 8A and 9B). DAF has four extracellular CCP domains, a highly glycosylated domain and a GPI anchor (160). DAF inhibits the AP C3 convertase by binding to Bb on the vWA domain with its CCP2 domain (161). CCP2 of DAF has a higher affinity for Bb compared to the intact FB. As a consequence, the active convertase is more sensitive to rapid decay, compared to C3bB (162, 163). DAF also binds to the C3 convertase on C3b via its CCP4 domain (163) and CCP-3 contributes to the accelerated decay function (164).

#### Factor H – The Master Regulator of the Alternative Pathway

Factor H regulates the AP and the amplification loop of the complement pathways (Figure 9). It is a soluble inhibitor of the C3 convertase competing with FB for binding to C3b (Figure 1A) (165). It also serves as a cofactor for C3b inactivation by FI (Figure 9A) and induces C3bBb complex dissociation (Figure 9B) (166, 167). FH is composed of 20 CCP domains arranged as beads on a string (168) (Figure 9C). CFHL1 is a shorter protein, containing the seven N-terminal domains of FH, generated via alternative splicing from the same gene (169) and functions as a fluid phase complement regulator. The fluid phase convertase C3(H_2_O)Bb is more resistant than the alternative cell-bound convertase and is less susceptible to regulation by FH (114). FH has two main ligands, C3b and the GAG, found on host cells surface. Recent crystallographic and mutation analysis resulted in precise mapping of the C3b and GAG-binding sites in FH.

Factor H binds to C3b and C3(H_2_O), but not to uncleaved C3. The conformational changes in the TED and CUB domains accompanying the transition from C3 to C3b expose the FH-binding sites. FH binds to C3b in at least two regions, at the N-terminus and the C-terminus of the protein. The N-terminal four CCPs contain the complement regulatory activity (132) and the CCP1–4 interacts with the MG ring of C3b. CCP-3 interacts with the CUB domain and CCP4 interacts with the TED domains (132, 142). CCP1–2 competes with FB causing its dissociation from C3b (Figure 9C). Both Bb and CCP1–2 are negatively charged leading to electrostatic repulsion. CCP19–20 carry a second binding site for C3b and can also interact with C3d (130, 131, 170, 171).

It has been difficult to unravel questions regarding the stoichiometry and architecture of the C3b–FH complex in the physiological fluid phase and on the surface of cell (Figure 9C). Does FH interact in the same way with C3b and C3(H_2_O) or with C3b in fluid phase and on cell surface? Could FH bind with its N- or C-terminal domains on the same C3b molecule or can it interact with two adjacent C3b or C3b and C3d molecules or with one C3b molecule via CCP1–4 at the C-terminus and engage in cell membrane binding? If it is assumed that FH CCP1–4 and CCP19–20 bind to the same C3b molecule and if there is a liner arrangement of the C-terminal CCPs, could there be a steric clash between CCP18 and CCP4? The crystal structure of CCP18–20 indicates a bend-back conformation of CCP18, allowing binding on the TED domain of both CCPs 1–4 and CCPs 19–20 (172). Studies of the C3b/C3d binding site on CCP19–20 showed that it may be overlapping, but it is not identical with the GAG-binding site (173–177). The C3b/C3d-binding site is extended toward the CCP19 and the GAG binding is extended toward the apex of the CCP20. It is possible that FH19–20 domains may bind both C3d and GAG at the same time (131, 172, 174, 177). Site-directed mutagenesis of the FH19–20 domains indicates that a ternary complex between C3d/FH19–20/heparin can be formed and is essential for the functional activity of FH (177). The formation of a ternary complex was confirmed by the crystal structure of FH19–20 with C3d and a model sialylated trisaccharide, where a surface area extending from SCR19 to the beginning of CCP20 binds C3d and CCP20 carries a is highly conserved binding site, which may accommodate GAGs and sialic acid containing glycans (174). Structural analysis of the complex of FH19–20 with C3d showed that CCP20 also may interact with C3d suggesting potential competition between C3d and GAG at this site of FH (130). Analysis of published PDB files indicates that this CCP20–C3d interaction is present in the other FH CCP19–20 crystals, but was considered a crystallization artifact. Nevertheless, mutations in CCP20 appeared to affect the interaction with C3b and C3d, suggesting that a C3d–CCP20 interaction is possible. Based on the accumulating structural and functional data, it can be hypothesized that the architecture of the C3b–FH complex is governed by the target surface and the density of the C3b and C3d molecules (Figure 9C). On host cells, one isolated C3b molecule will bind CCP1–4, while SCR7 and the C-terminus will interact with the GAG of the cell membrane. Since FH may circulate in plasma in a folded back hairpin conformation (178–181), simultaneous interactions with the N- and C-termini to the same C3b molecule could be possible, while CCP7 and 20 bind to the GAGs of the membrane. Indeed, CCPs 10–13 are involved in the inclination of FH, allowing both CCP1–4 and CCP19–20 binding to C3b (182). Using crystal structures of CCP10–11, CCP11–12, and CCP12–13, the authors also demonstrated that a tilt of 80–100° occurs allowing a hairpin structure formation. The compact architecture of the C3b–FH complex is supported by the existence of a third binding interface involving CCP6–10 in FH and the C3c portion of C3b (171, 183, 184).

Glycosaminoglycans are an important constituent of the cell membrane and play a critical role in complement regulation. In addition to the GAG-binding site in CCP20, FH carries another GAG-binding site located in CCP7 (183, 185–189). These GAG-binding sites in FH allow it to recognize negatively charged heparan sulfate moieties on the membrane and may explain the differences in the affinity of FH binding and a dependence on the expression of GAG and sialic acid on the cell surface (190). The difference in the susceptibility of sheep and rabbit erythrocytes to lysis by human complement, which are at the basis of the classical hemolytic tests for complement activation, is expression level of sialic acid on the surface of these cells. This allows them to bind (sheep) or not to bind (rabbit) to FH (165, 191). Together with the low affinity of properdin for heparin and heparan sulfate (94), GAG expression is a powerful regulator of the complement homeostasis between negative regulation and stimulation of the complement pathways. The two regions anchoring FH to the cell membrane, CCP7 and CCP20, are specialized in binding to unique GAG, expressed in different types of cells, however both are necessary to assure functional activity of FH on the cell surface (189). CCP7 containing construct CCP6–8 binds stronger to heparin than CCP20 containing construct CCP19–20 (192), while CCP6–8 and CCP19–20 do not recognize the same sulfate GAG. GAG and sialic acid are expressed in multiple human organs with different subpopulations and distinct structures that may provide a variation of the binding affinity of complement regulators (193). These differences can potentially explain why polymorphisms or mutations in these regions are associated with complement-mediated diseases.

Mutations or polymorphisms in the GAG-binding sites of FH may create an imbalance in the homeostasis of complement regulation and could explain its association with different diseases. For example, a polymorphism in CCP7 leading to a modification from a tyrosine to histidine at amino acid 402 (Y402H) is the strongest genetic susceptibility factor for age-related macular degeneration (AMD) (194–197). Detailed analysis of this mechanism revealed that CCP7 in FH binds not only to GAG, but also to oxidized lipids, including malondialdehyde (MDA) (198–200). Y402H binds more weakly to MDA and oxidized phospholipids expressed on retinal pigment epithelium compared to the non-mutated protein (198, 200).

Another ligand of CCP7 and CCP19–20 in FH is the CRP, which is secreted by the liver during inflammation acute phase. Binding of FH to CRP can enhance complement inhibition, particularly on apoptotic or damaged cells during inflammatory conditions (201).

**Figure 10:**
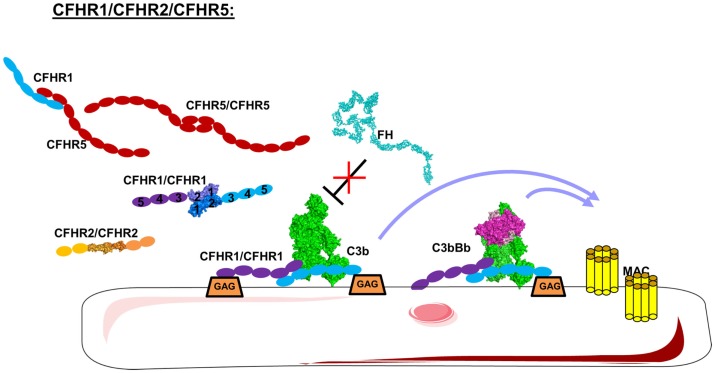
**Domain organization and mechanism of CFHRs**. CFHR1, 2, and 5 carry dimerization N-terminal domains allowing them to form homo and heterodimers. These CFHRs, particularly CFHR1, are downregulators of FH, competing with FH for C3b and binding on the cell surface. This allow C3 convertases and MAC formation.

The interaction of FH with different cell surfaces is controlled by CFHR proteins. CFHRs belong to the FH family and comprise five different members (CFHR1–5). CFHRs are composed of five to nine CCP domains, present a high sequence homology with FH, and are recently described in detail (202). CFHR1, CFHR2, and CFHR5 share high homology in their two N-terminal domains, allowing them to form homo- and heterodimers (Figure 10) (203, 204). CFHR3 and CFHR4 do not form dimers, the C-termini of each share a high sequence homology with FH leading to competition between CFHRs and FH for binding to C3b, C3d, and GAG on the cell surface (203, 205, 206). Therefore, the CFHRs will enhance complement activation, preventing the action of FH.

## C5 Convertases and the Terminal Complement Pathway

**Figure 11:**
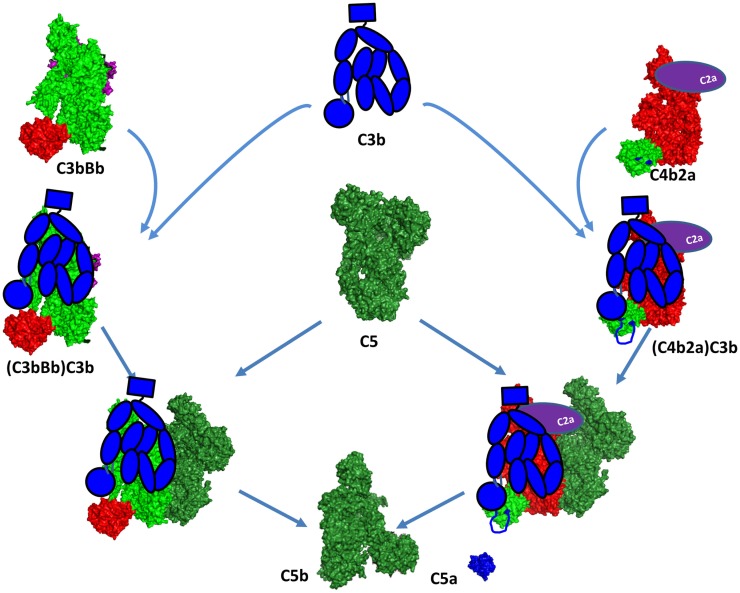
**C5 convertase formation**. A C5 convertase is generated when a C3b molecule binds covalently in the vicinity or directly to a C4b or C3b, already engaged in a C3 convertase. This new enzyme loses its capacity to cleave C3 and starts to cleave C5. The binding site of the second C3 molecule is unclear, but it has been suggested to bind to the TED domain of C4d and to the CUB domain of C3b. Since the atomic coordinates of the two C5 convertases have not been published yet, this figure represents the current model for their organization. The CP C3 convertase is modeled here on the basis of the structures of C4b, C2a, and the AP C3 convertase C3bBb. The second C3b molecule is depicted in a schematic representation in blue to be distinguished from the C3b molecule interacting with FB to form the C3bBb complex. The Bb and C2a fragments are depicted in magenta and violet, respectively. They are partially visible behind the C3b and C4b molecules.

**Figure 12:**
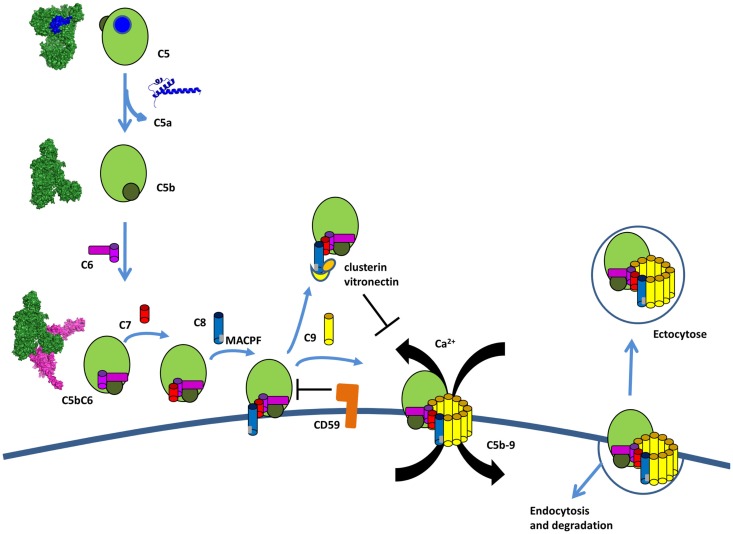
**The terminal complement pathway**. The C5 convertase cleaves an inert molecule of C5 into a potent anaphylatoxin, C5a, and a bioactive fragment C5b. C5b interacts with C6, C7, C8, and multiple copies of C9 to form the membrane attack complex C5b-9 (MAC). C5b-8 inserts into the membrane and C9 polymerize to form a pore inducing Ca flux and pathogen lysis. Host cells are protected from lysis by expression of CD59, which prevents the insertion and by clusterin and vitronectin, which bind to C8 and prevents insertion in the membrane. If MAC is bound to the membrane, host cells can internalize it or remove it by ectocytose.

C3b binds to the C3 convertase to form a new enzymatic complex – C5 convertase which cleaves C5 to bioactive fragments C5a and C5b (Figure 11). C5b recruits complement components C6, C7, C8, and C9 which polymerize to form the membrane-attack-complex (MAC) ring (Figure 12) (207). The structures of individual components and overall architecture of the C5b-9 complex are starting to be elucidated, while, the structure and the mechanism of action of the CP and AP C5 convertases is not fully understood and require further investigation.

### C5 convertases

The C3 convertases C4b2a and C3bBb are the precursors of the C5 convertases. The C3 convertases are able to bind C5, but with a very low affinity and cleavage rate. These C3 convertases switch their specificity and start to bind and cleave C5 only after binding of an additional C3b molecule in the immediate proximity or most likely over the C3 convertase itself (Figure 11) (208). The structure of this trimolecular complex is unknown, but has been suggested that the covalent C3b-binding site on C4b is the residue Ser1217 (p.Ser1236) within the TED domain (209). This residue is not conserved in C3b and the binding site seems to be located outside of the TED domain, in the α43 fragment (residues 1268–1641) (210), suggesting different topology of the two trimolecular complexes. Whether the newly bound C3b molecule interacts with C5 or affects the conformation of the C3 convertase subunits is currently unknown. Laursen and colleagues propose a model by which the MG1, MG4, MG5, and TED domains of C3b will be able to contact the CUB and TED domains of C4b. The CUB, MG2, MG6, and MG3 domains appear to be capable of reaching mainly the rest of C4b, while the MG7, MG8, and C345C domains potentially could be in direct contact with C5 (Figure 11) (211).

The dramatic conformational change of C4 upon release of C4a is very similar to C3 (212). TED domain, MG8, and CUB are exposed after C4 cleavage, allowing covalent bond with Gln1013. The crystal structure of C4b confirms the implication of the flexibles residues 1231–1255 in the interaction with the TED domain of C3b (209, 212). This model supports the idea that C3b has no direct interaction with C5 in the classical (and most likely in the alternative) C5 convertase (Figure 11). C4b and CVF-C5 structures suggest that C4b-binding area for C5 is within the domains MG4, MG5, and MG7, supporting Laursen hypothesis.

The loss of affinity to C3 and the acquisition of affinity to C5 results in cessation of C3b opsonization and initiation of MAC-induced membrane damage. Upon covalent binding of a C3b molecule to a CP convertase C4b2a, there is a formation of a trimolecular complex with about 1000-fold increased enzymatic activity toward C5 cleavage (213) compared to the bimolecular complex. The CP C3 convertase cleaves on average 1.5 C5 molecules per minute. The gained activity of the trimolecular complex of the AP C3bBbC3b is six to ninefold weaker compared to the classical, cleaving less than 0.3 C5 molecules per minute (214). To compensate for the weaker efficacy, the AP C5 convertase can be stabilized by properdin (215, 216), which increases its half-life from approximately 3–30 min. This is different than the classical C5 convertase, for which a natural stabilizer has not been described. Together with the fact that the AP amplification loop generates a large number of C3b molecules makes the AP C5 convertase the main source of the terminal pathway complex C5b-9. In 2002, Pangburn and Rawal proposed a ring model for the amplification of the complement activation on the cell surface (213). In this model, any deposited C3b molecule coming from the classical or AP initiation can form an AP C3 convertase that will cleave one or several molecules of C3 (depending on the stabilization by properdin). A ring of new C3 convertases is formed or C3b binds on top of the C3 convertase itself to form a C5 convertase. As the activation spreads, the older enzymes will be inactivated by FH and FI leaving an expanding inner core of inactivated C3b surrounded by an active band of C5 convertases. The outermost band of newly formed C3 convertases is responsible for the growth of the site as it expands across the surface of the target.

#### Complement-Independent Cleavage of C5 and C3

Accumulating evidence suggests the existence of additional complement activation pathways in the plasma (217) aside from the three established pathways. Thrombin, human coagulation factors IXa, XIa, Xa, and plasmin were all found to effectively cleave C3 and C5 (218). C5a and C5b can be generated from C5 via thrombin, independently of the plasma complement system (217, 219). Thrombin cleaves C5 poorly at R751, which is the site of action of the C5 convertase (220). However, it does cleave C5 at an alternate site, R947, generating intermediates C5T and C5bT. These activation fragments will be generated at sites of activation of the coagulation cascade. Interestingly, C5bT formed a C5bT-9 MAC with significantly more lytic activity than with C5b-9.

Complement-independent cleavage of C3 by plasmin has also been suggested in the literature (221–223). The fragments generated by plasmin-mediated cleavage (C3a-like, C3b-like, iC3b-like, C3c-like, and C3dg-like) are similar, but not identical to fragments generated by the complement cascade and are biologically active.

### Membrane-attack-complex C5b-9 formation

Activation of the terminal complement pathway results in formation of MAC that form large, 10 nm wide, pores in the target membrane (207). These complexes are formed when C5 is cleaved into C5b by the C5 convertase. Upon cleavage, C5b undergoes a dramatic conformational change, similar to C3b, but with a TED domain ending up only halfway the distance to the MG ring (Figure 12) (224). This conformation of C5b interacts with C6, which wraps around the TED domain of C5b. C6 to C9 are homologous proteins that share similar central membrane-insertion domains called MACPF. Binding of C7, C8, and multiple copies of C9 results in MAC formation with C5b–C6–C7–C8β–C8αγ–C9 making an arc with a protrusion at the beginning by C5b. C5b-7 is lipophilic and binds to the cell membrane (225). C8 is the first component to penetrate the lipid bilayer upon interaction with the forming MAC. The structure of the MACPF domain of C8α (226) shows homology to perforin, a pore-forming protein released by cytotoxic T and NK cells, as well as, to bacterial cholesterol-dependent cytolysins. This resemblance suggests a common membrane perforation mechanism for MAC, perforin in the mammalian immune system, and bacterial pore-forming proteins (226). A single MAC contains up to 18 C9 molecules forming a tubular channel. However, only one or two C9 molecules are sufficient to form functional pores (227, 228).

Each functional MAC is sufficient to lyse by colloid osmosis in the membrane of metabolically inert cells, like erythrocytes or liposomes (229). Gram-negative bacteria are also susceptible to complement killing, in particular the meningitis causing *Neisseria* species (230, 231). Individuals deficient in terminal complement components are at increased risk for recurrent meningitis. Gram-positive bacteria have an extremely thick cell wall that MAC cannot penetrate leaving them resistant to complement elimination. Metabolically active nucleated cells are also resistant to lysis by complement (228, 232). In order to induce killing in these cells, multiple MACs must be inserted in the cell membrane together with coordination of calcium flux and not well-understood signal transduction (233). Once MACs have inserted in these cells, calcium flux is induced in the pore from the extracellular space or is released from intracellular stores (234). Subsequently, multiple still not well-known signaling pathways are activated leading to cell proliferation or apoptosis, which is dependent on the targeted cell type and experimental conditions.

### Membrane-attack-complex regulation

Membrane-attack-complex formation is tightly regulated to avoid accidental host cell damage and activation (Figure 12). C8 was suggested to play a dual role in MAC formation and regulation. In the absence of a cell membrane, the binding of C8 to C5b-7 induces conformation changes that result in a loss of ability to form pores, causing it to act as a MAC inhibitor (235). If soluble dead-end products sC5b-7, sC5b-8, and sC5b-9 are generated in fluid phase and do not bind to a membrane, they are scavenged by clusterin and vitronectin. These two regulators bind below the C5b-9 arc rendering it water soluble and preventing membrane binding (224, 236–238).

The GPI anchored protein, CD59 is expressed on most cell types (239, 240) and blocks membrane perforation by C5b-8 and C5b-9 (241, 242). CD59 does not bind free C8 and C9, but does interact with the MACPF domain of each protein upon conformational changes associated with C5b-9 complex formation (243–245). Furthermore, the lytic terminal complex of complement C5b-9 can be removed within minutes of its deposition on the membrane of target cells either by shedding via membrane vesicles (exocytosis) or by internalization and degradation (246–249).

## Complement Anaphylatoxins

The anaphylatoxins, C3a and C5a, are constantly released during complement activation. These small (10–14 kDa) peptides play a critical role in supporting inflammation and activation of cells that express anaphylatoxin receptors (250). To enhance inflammation, anaphylatoxins recruit immune cells to the site of complement activation and induce oxidative bursts on macrophages (251, 252), eosinophils (253), and neutrophils (254, 255). However, some studies challenged the concept for the pro-inflammatory role of C3a. C3a has a more complex function, depending on the context, with a balance between pro- and anti-inflammatory roles. The highlight anti-inflammatory properties of C3a re-evaluate its physiological role during inflammation (256). Furthermore, C3a and C5a induce histamine production by basophils (257, 258) and mast cells (259) to provoke vasodilatation. C5a also recruits T-cells (260) and myeloid-derived suppressor cells (261) that constitutively express C5aR. Although the functional activity of C4a is debated, it has been reported to activate macrophage and monocytes (262, 263). However, a lack of cognate C4a receptor identification and unreproducible data (264) warrant further studies to determine the physiological role of C4a.

**Figure 13:**
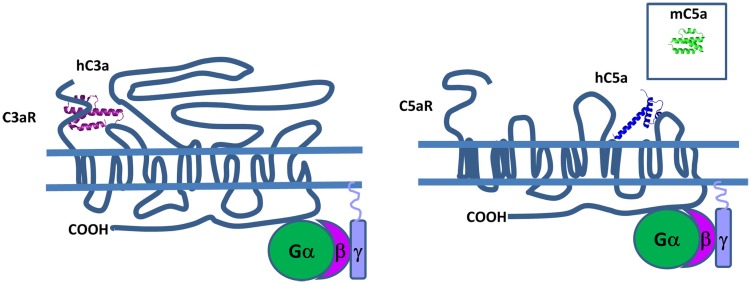
**Complement anaphylatoxins**. C3a and C5a have a four and three helical bundle topology. Mouse C5a (in the square) is different from its human counterpart, because it has four helical bundle structure. These anaphylatoxins bind to G protein-coupled receptors C3aR and C5aR and stimulate pro-inflammatory signaling pathways.

Structural data show that both human C3a and C5a adopt an alpha-helical conformation with four- and three-helical bundles, respectively (Figure 13). The C5a crystal structure has a core domain constituted as an antiparallel alpha-helical bundle and the C-terminal domain links the core domain by a short loop containing two adjacent arginines in position 62 and 74 (265) that both interact at the same binding site on the receptor. In human plasma, these two fragments are rapidly converted by carboxypeptidase N to C3a desArg and C5a desArg by cleavage at the C-terminal arginine (266, 267). C3a desArg has a very similar structure to C3a (268), but is incapable of binding to C3aR. The alpha1-helix of C5a desArg is detached at the three others alpha-helices (269). Due to this conformational change, human C5a desArg has 90% weaker pro-inflammatory activity compared to C5a (270). In contrast, murine C5a desArg is as potent as the murine C5a upon binding to C5aR on murine cells (271), which could be explained by the lack of major structural changes in the murine C5a desArg, compared to C5a. Both murine proteins form a four-helix bundle, contrary to the human C5a desArg, which adopts a three-helix bundle conformation upon cleavage of the terminal Arg residue. These inter-species differences need to be taken into account during analysis of *in vivo* experiments.

C3aR, C5aR, and C5aL2 belong to the G protein-coupled receptor (GPCR) family that contain seven transmembrane domains that are able to interact with C3a and C5a (Figure 13). The C3a-binding site of C3aR is located in the large second extracellular loop that contains a sulfotyrosine 174, which is critical for C3a docking (272). This interaction induces phosphorylation of intracellular pathways including PI3K, Akt, and MAPK leading to chemokine synthesis (273).

There are two sites in C5aR that are essential for C5a binding. The first sight consists of basic residues from human C5a that interact with sequences rich in aspartic residues located on N-terminal extracellular domain of C5aR (274). The second site is in a binding pocket located near the fifth transmembrane domain (275) and interacts with the C-terminal region of human C5a (276). Then, two distinct clusters of hydrophobic residues allow a molecular switch in C5aR leading to G protein activation (277). This mechanism exposes preserved residues clustered in two intracellular and two transmembrane domains that participate to the initial interaction with G proteins (278). The second extracellular loop plays a role of a negative regulator of C5aR activation and may stabilize the inactivated form of the receptor (279). By binding human C5a, C5aR induces downstream effects including activation of PI3K-γ (280, 281), phospholipase Cβ2 (282), phospholipase D (283), and Raf-1/B-Raf-mediated activation of MEK-1 (284).

Human C5a and C5a desArg are also able to bind C5L2 (285). C5L2 is again composed of seven transmembrane domains, however it is not coupled with G protein due to an amino acid alteration at the end of the third transmembrane in the DRY sequence (286). C5a has lower affinity to C5L2 compared to C5a desArg (287) and recently it has been demonstrated that C5L2 is a negative regulator of anaphylatoxin activity (288). It has also been reported that C5L2 and C5aR form a heterodimer (289) and this complex induces internalization of C5aR upon C5a binding (290). This internalization is essential for the induction of the late stage of ERK signaling (291, 292).

## Complement Receptors for C3 Activation Fragments

Complement participates actively in the opsonization of pathogens and dying host cells, in addition to the clearance of immune complexes. Recognition molecules in the CP and LP, as well as cleavage fragments of C3, opsonize the target structure and serve as bridging molecules with receptors on the surface of the phagocytes. Depending on the type of the opsonin present (C3b, iC3b, or C3d), the phagocyte will generate a pro-inflammatory response or tolerogenic suppression.

**Figure 14:**
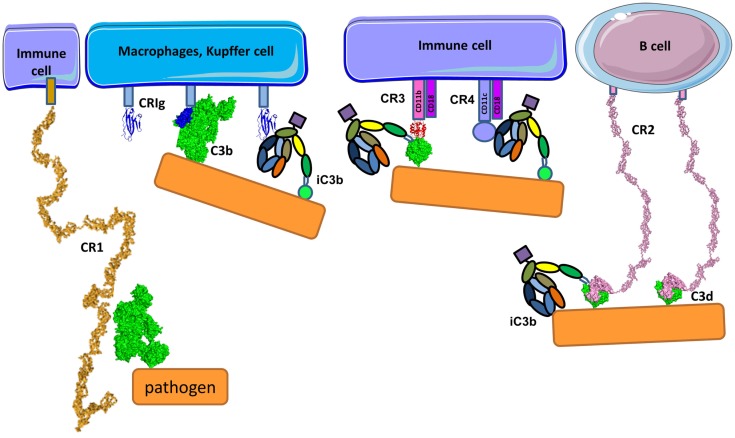
**Complement receptors**. CR1 is composed of CCP domains and is expressed primarily by immune cells and erythrocytes. Apart from being cofactor of FI, CR1 is also a complement receptor facilitating immune complex clearance and phagocytosis. CR1 interacts with C3b. CRIg has immunoglobulin-like structure in its C3b recognition domain. CRIg binds to C3b and iC3b and is expressed on macrophages and Kupffer cells. Immune cells also express CR3 and CR4 containing integrin domains that bind to iC3b (and C3d for CR3) on different binding sites on iC3b molecule. CRIg, CR3, and CR4 facilitate phagocytosis and modulate the activation state of cells. CR2 is expressed primarily on B-cells and recognizes C3d using the first two CCP domains. It serves as a co-stimulatory molecule for the B-cell receptor upon binding C3d-opsonized pathogen.

Pathogens, immune complexes, and cell debris opsonized by C3 cleavage fragments can be recognized by CR (Figure 14) with three different structural organizations: containing CCP modules (CR1 and CR2), integrin family members (CR3 and CR4), and the immunoglobulin superfamily member (CRIg) (293). CR1 is expressed on monocytes, macrophages, neutrophils, erythrocytes, and renal podocytes, CR2 is found on B-cells, CR3 and CR4 are expressed by macrophages, monocytes, dendritic cells, neutrophils, and NK cells and CRIg has restricted expression and is found mainly on Kupffer cells in the liver and resident tissue macrophages (294). Interestingly, the expression of CRIg on macrophages in inflamed tissue is lower compared to macrophages outside of an inflammatory area (295).

### Complement receptor 2

Complement receptor2 (CD21) is expressed on B-cells interacting with C3d and iC3b on the surface antigens (Figure 14) forming a co-receptor complex with CD19 and CD81 (296). C3d serves as a molecular adjuvant by lowering the threshold for B-cell activation by 1000–10,000-fold (297). The TED domain of C3 has a completely different conformational environment in the native protein as compared to its degradation products C3b, iC3b, C3dg, and C3d. Recent studies showed that the binding site for CR2 in iC3b and C3d lies within the common TED domain (298). The C3d-binding site is located in the two N-terminal CCP domains of CR2 (299). Two different crystal structures had been proposed for the complex CR2:C3d; the first one, described in 2001 by Szakonyi et al. (300), showed that only the CCP2 domain of CR2 interacts with C3d. Contrary to this result, biochemical studies showed that mutations on several basic residues on CCP1 domain affected C3d binding to CR2 (301). In 2010, a second structure was proposed (302) in agreement with the mutagenesis data (301) where both CCP1 and CCP2 are involved in the interaction (Figure 14). One possible explanation for the discrepancy between structures could be due to the high concentration of zinc in the crystallization buffer from 2001 leading to a non-physiological complex.

### Integrin family complement receptors CR3 and CR4

Integrin family CR3 (also known as CD11b/CD18, α_M_β_2_ or Mac-1) and CR4 (also known as CD11c/CD18 or α_X_β_2_) are heterodimeric transmembrane complexes, composed of a unique α-chain and a common β-chain. They bind multiple ligands participating in phagocytosis, cell adhesion to the extracellular matrix, leukocyte trafficking, synapse formation, and co-stimulation. Ligand binding and signaling through integrin receptors is governed by a complex cascade of conformational changes, known as inside-out signaling (303). A receptor in its inactive, bend-closed conformation can respond to a cytoplasmic signal, transmitted inside-out through the β-chain, passing to a low affinity binding extended-closed and high affinity binding extended-open conformation. Upon ligand binding, another signal is transmitted outside-in, leading to raid cellular response, including actin remodeling, phagocytosis, degranulation, or slow responses involving protein neosynthesis. CR3 and to lesser extent CR4 are essential for phagocytosis of C3 fragments, opsonized immune complexes, and pathogens (304). CR3 and CR4 differ in their profile of recognized C3 fragments because both receptors bind to iC3b, but CR3 recognizes C3d, while CR4 binds to C3c, suggesting that the two receptors have distinct binding sites on the iC3b molecule (Figure 14) (305, 306). The iC3b and C3d binding site of CR3 is located in the VWF-A domain of the α-chain, also called αI domain, and binds in a divalent metal ion-dependent manner (307). This type of domain is present also in FB, which requires divalent ions (Mg^2+^) in order to interact with C3b. However, the two VWF-A containing molecules do not bind to the same fragments of C3b (113, 305). FB interacts only with C3b, while CR3 binds to iC3b and C3d and the binding sites for these VWF-A domains are distinct (Figure 14). VWF-A domain of CR3 binds to the TED domain (C3d part) of iC3b and the CUB and C345C domains of iC3b may also contribute to the interaction with the β-propeller and βI domains of the α-chain of CR3 (305).

In contrast to CR3, CR4 binds to the C3c portion of iC3b (Figure 14). The architecture of the complex has only been observed at low resolution, by electron microscopy and displays a CR4 binding site at the interface between MG3 and MG4 (306). The flexibility of the CUB domain after cleavage by FI allows re-orientation of the C3c part of iC3b leading to better exposure of the MG3–MG4 interface (9, 308). Initial studies of the structure and molecular details of the complex conformational changes in CR4 are starting to be elucidated (309) showing that CR4 binds iC3b through the αI domain on the face known to bear the metal ion-dependent adhesion sites similar to CR3 (306).

### Immunoglobulin superfamily receptor CRIg

Immunoglobulin superfamily receptor CRIg is a CR expressed on macrophages and Kupffer cells in the liver that binds to C3b and iC3b (Figure 14) and mediates the phagocytosis of opsonized particles and pathogens (310, 311). CRIg acts as an inhibitor of the AP preventing the entry of the substrate molecule C3 and C5 into the C3 convertase (312). The CRIg-binding site on C3b is located in the MG ring of the β chain, engaging MG3 and MG6 domains. A conformational change in MG3 during the transition of C3 to C3b contributes to the formation of the CRIg-binding site and explains why CRIg binds to C3b and iC3b, but not to intact C3.

## Understanding Complement-Related Diseases Using Structure–Function Relationships

The importance of the complement system in physiology is illustrated by the severe and life threatening diseases occurring due to inefficient or excessive complement activity. Abnormal complement activity is associated with many inflammatory, autoimmune, neurodegenerative, and age-related diseases. Here, we will describe the role of complement dysfunction in aHUS.

### Atypical hemolytic uremic syndrome

**Figure 15:**
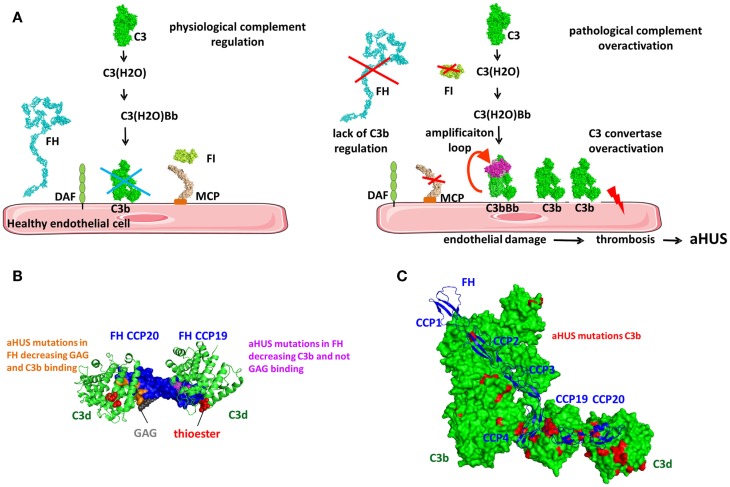
**Understanding aHUS using structure–function relationships**. **(A)** The role of complement alternative pathway in the physiopathology of aHUS. On healthy endothelial cells, deposited C3b is rapidly inactivated by regulatory molecules including FH, MCP, and FI. For FB binding and C3 convertase formation, FB is dissociated by FH and DAF preventing excessive host tissue damage. Mutations in the complement regulators FH, MCP, and FI can result in inefficient complement regulation. Mutations in the components of the C3 convertase (C3 and FB) induce the formation of overactive C3 convertase or a convertase that is resistant to regulation. In both cases, the complement cascade is activated on glomerular endothelial cell surface leading to endothelial damage, thrombosis, erythrocyte lysis, and aHUS. **(B)** FH mutations in the CCP19–20 region are mapped on the structure of the C3d–C3d–FH19–20 complex. A model GAG bound to FH CCP20 is indicated in gray. FH disease-associated mutations that decrease only C3b-binding are indicated in orange and mutations decreasing both C3b and GAG binding are in magenta. Reduced C3b and/or GAG binding will cause inefficient endothelial cell protection and complement overactivation. **(C)** C3 mutations found in aHUS patients. The majority of the mutations (in red) are not randomly distributed, but mapped to the FH binding sites on C3b. These mutations correlated with decreased FH and MCP binding allowing characterization of the MCP binding site, which overlaps with the FH binding site in CCP-3–4.

The aHUS is a rare thrombotic microangiopathy that predominates in the kidney. Without appropriate treatment, it leads to end stage renal disease in approximately 60% of patients (313, 314). This thrombotic microangiopathy is different than typical HUS and thrombotic thrombocytopenic purpura because it is not associated with infection by Shiga toxin-producing bacteria or ADAMST13 deficiency, respectively. aHUS occurs at any age and has a poor prognosis (prior to the development of Eculizumab). In contrast, typical HUS is predominantly a pediatric disease and has a favorable renal outcome. aHUS is characterized by a triad of hemolytic anemia due to fragmented erythrocytes, thrombocytopenia, and acute renal failure. Renal failure is a result of platelets rich microthrombi, formed in the small vessels (capillaries and arterioles) of the kidney resulting in a prothrombotic state. The hallmark of the aHUS is the association with alternative complement pathway mutations (Figure 15A). Endothelial damage is known to be related to complement dysregulation. Screening for and characterization of mutations in the components of the C3 convertase (C3 and FB), its regulators (FH, FI, MCP, CFHR1, and thrombomodulin), or anti-FH antibodies has become an indispensable part of the diagnostic of the disease (315).

The importance of screening for mutations in complement factors can be observed in the examples of FH and C3. The majority of the mutations in FH found in aHUS patients does not induce protein quantitative deficiency and are located in the C-terminus of the protein (CCP19–20). These mutations affect either the interaction with C3b, GAG, or both ligands leading to impaired cell surface protection against complement attack [(175, 177, 316); summarized in Ref. (191, 315, 317, 318)]. Mapping of these disease-associated mutations on the complex of C3d with FH19–20 (130, 131, 174) revealed that the residues which decreased C3b-binding mapped to the C3b/C3d-binding site of FH in CCP19. The residues that affected both C3b and GAG interaction were located in the FH CCP20 interface (Figure 15B). These structural analyses help to explain the mechanism by which genetic abnormalities in FH induce impairment of C3b and/or GAG binding and hence a predisposition to develop aHUS. A similar phenomenon is observed with C3 mutations found in aHUS. These genetic changes are not randomly distributed but cluster alongside FH CCP1–4 and CCP19–20 binding sites (Figure 15C) (142, 315, 319, 320). Functional analysis revealed that mutations located in the FH-binding sites resulted in decreased FH binding, thus showing the link with aHUS. Currently, there is a debate as to whether or not in physiological conditions FH CCP20 can interact with an adjacent C3d molecule (130, 131, 174). It is possible the observed interaction between the second molecule C3d and FH CCP20 may be a crystallization artifact and in turn the functional consequences of certain aHUS associated FH and C3 mutations will be more difficult to explain. However, these mutations fall within this suggested C3b/C3d binding site and indeed decrease FH/C3b interaction. This case exemplifies how structural analyses can aid in understanding disease physiopathology and how disease physiopathology improves our understanding of complement.

Disease-associated mutations also has resulted in the mapping of the MCP binding site on C3b. C3 mutations that decreased MCP binding mapped in an area overlapping with the FH CCP-3–4-binding site (142). However, these mutations did not affect CR1 binding suggesting that the CR1 binding site is distinct and that CR1 is not involved in aHUS (142). Experimental and structural analysis revealed that for FH and C3 almost all studied genetic changes mapped to the ligand binding site and had clear functional consequences. aHUS mutations in FB seem to be exception of this rule. Mutations in FB were located in multiple domains of the protein and in more than half of the cases they were far from any known binding sites (321). Mutations located within the C3b-binding site did induced formation of an overactive C3 convertase or a convertase resistant to regulation (321–323). The mutations far from this binding interface showed no functional defect as observed by FB functional tests (321). As shown for the majority of complement mutations in aHUS, mapping of disease-associated mutations together with detailed functional analysis should be performed to understand the mechanism of complement dysregulation associated with a disease.

### Structural basis of therapeutic complement intervention

We have described in depth the known molecular mechanisms of the complement system and this unleashes many possibilities for rational design of complement inhibitors for treatment of disease (324, 325). Here, we will give a few examples that illustrate this concept. Blockade of the late effector functions of complement can be obtained if the cleavage of C5 by the two C5 convertases is prevented. The therapeutic monoclonal antibody, Eculizumab, targets human C5 and blocks cleavage by C5 convertases (326). The Eculizumab binding epitope on C5 interacts with the contact interface between C5 and the C5 convertase (211) preventing the entry of C5 into the C5 convertase and blocking further cleavage and generation of the bioactive fragments C5a and C5b-9. Eculizumab blocks the terminal complement pathway but leaves the C3 convertases unaffected. Eculizumab showed significant improvement in clinical outcome and has been accepted for treatment of complement-mediated diseases including paroxysmal nocturnal hemoglobinuria (PNH) (327) and aHUS (328, 329) and clinical trials are ongoing for other diseases.

In order to control complement at an earlier step, inhibitors acting at the level of C3 have also been designed. Compstatin is a 13-residue peptide that is being tested pre-clinically. It binds to C3 and blocks its cleavage (330, 331). Compstatin binds to MG4 and MG5 of C3c and C3b where it undergoes a large conformational change upon interaction (332) causing steric hindrance of the substrate C3 to the convertase complexes and blocking complement activation and amplification. Compstatin has shown efficacy in complement blocking *in vitro* and in animal models including extracorporeal circulation (333), sepsis (334), and PNH (335). Both Compstatin and Eculizumab are species-specific and act only in humans and monkeys, but not in mice or rats, reflecting subtle differences in the structure of human and murine complement components.

Another strategy of rational design of complement inhibitors is to target the regulatory domains of FH or CR1 to the cell surface via potent C3b, iC3b, C3d, or membrane recognition domains, derived from FH or CR2. Mini FH molecules, containing CCP1–4 and CCP19–20 bind C3b and C3d with high affinity and show better efficacy compared to native FH in *in vitro* models of aHUS (336) and PNH (337). A hybrid molecule TT30, containing FH CCP1–5 and CR2 CCP1–4 is designed to accumulate preferentially at sites already under complement-mediated attack (338). TT30 interacts simultaneously with C3b and C3d merging the functionality of fluid phase FH binding to C3b with CR2 interaction to C3d on the surface of host cells (339). TT30 and its murine analog showed significant improvement in models of AMD, ischemia/reperfusion injury, and PNH (340–342).

## Conclusion

Currently, we know that complement is not only a simple lytic system, but rather a powerful innate immune surveillance tool, serving as a sentinel against pathogens, modulator of the adaptive immune response, and as a regulator of host homeostasis. This cascade of enzymatic reactions is driven by conformational changes induced after a recognition event assuring that complement will be activated only when and where needed. This special and temporal control of complement activation is guaranteed also by the high specificity and selectivity of the enzymatic reactions, where the involved enzymes cleave only a single substrate and have a single ligand-binding site. In contrast to this high specificity of the propagation of the chain reaction, complement activation relies on target patterns binding by versatile recognition molecules, such as C1q, MBL, ficolins, and properdin. The activation of the three complement pathways leads to the generation of C3b, the Swiss army knife of complement, which interacts with a large variety of ligands and receptors with multiple distinct binding sites. The balance of these interactions determines whether full-blown activation will occur by the amplification loop of the complement pathways, with a generation of one of the most potent inflammatory mediators C5a or the effect will be attenuated by the C3b breakdown cycle. Again, the attenuation relies in large part on the capacity of a versatile recognition molecule FH to discriminate between self and non-self and to stop the amplification loop. C3b and C3(H_2_O) may bind to the cell surface via proteins-platforms, like properdin and P-selectin. Expression/binding of these molecules accelerates the complement activation and anaphylatoxin generation. The anaphylatoxins increase the expression/binding of P-selectin and properdin contributing to a vicious circle of complement activation, enhancing local inflammation, thrombosis, and tissue damage.

The knowledge of these molecular mechanisms that has been accumulated during recent years has allowed for better understanding of complement-related diseases. It also opens up the possibility for a rational design of novel molecules with therapeutic potential to control steps in the complement cascade. Clinical application of the anti-C5 blocking antibody Eculizumab has already demonstrated that controlling complement can revolutionize the treatment of patients with overactive complement-mediated diseases.

## Conflict of Interest Statement

The authors declare that the research was conducted in the absence of any commercial or financial relationships that could be construed as a potential conflict of interest.

## References

[1] Ricklin D Hajishengallis G Yang K Lambris JD. Complement: a key system for immune surveillance and homeostasis. Nat Immunol (2010) 11:785–97. 10.1038/ni.1923 20720586 PMC2924908

[2] Kolev M Le Friec G Kemper C. Complement – tapping into new sites and effector systems. Nat Rev Immunol (2014) 14:811–20. 10.1038/nri3761 25394942

[3] Merle NS Noe R Halbwachs-Mecare L Fremeaux-Bacchi V Roumenina LT Complement system part II: role in immunity. Front Immunol (2015). 6:257. 10.3389/fimmu.2015.00257 PMC4443744 26074922

[4] Lachmann PJ Halbwachs L. The influence of C3b inactivator (KAF) concentration on the ability of serum to support complement activation. Clin Exp Immunol (1975) 21:109–14. 52423 PMC1538241

[5] Pangburn MK Schreiber RD Müller-Eberhard HJ. Formation of the initial C3 convertase of the alternative complement pathway. Acquisition of C3b-like activities by spontaneous hydrolysis of the putative thioester in native C3. J Exp Med (1981) 154:856–67. 10.1084/jem.154.3.856 6912277 PMC2186450

[6] Nilsson B Nilsson Ekdahl K. The tick-over theory revisited: is C3 a contact-activated protein? Immunobiology (2012) 217:1106–10. 10.1016/j.imbio.2012.07.008 22964236

[7] Isenman DE Kells DI Cooper NR Müller-Eberhard HJ Pangburn MK. Nucleophilic modification of human complement protein C3: correlation of conformational changes with acquisition of C3b-like functional properties. Biochemistry (1981) 20:4458–67. 10.1021/bi00518a034 7284336

[8] Li K Gor J Perkins SJ. Self-association and domain rearrangements between complement C3 and C3u provide insight into the activation mechanism of C3. Biochem J (2010) 431:63–72. 10.1042/BJ20100759 20666732

[9] Nishida N Walz T Springer TA. Structural transitions of complement component C3 and its activation products. Proc Natl Acad Sci U S A (2006) 103:19737–42. 10.1073/pnas.0609791104 17172439 PMC1750921

[10] Rodriguez E Nan R Li K Gor J Perkins SJ. A revised mechanism for the activation of complement C3 to C3b: a molecular explanation of a disease-associated polymorphism. J Biol Chem (2014) 290(4):2334–50. 10.1074/jbc.M114.605691 25488663 PMC4303685

[11] Winters MS Spellman DS Lambris JD. Solvent accessibility of native and hydrolyzed human complement protein 3 analyzed by hydrogen/deuterium exchange and mass spectrometry. J Immunol (2005) 1950(174):3469–74. 10.4049/jimmunol.174.6.3469 15749882

[12] Sahu A Kozel TR Pangburn MK. Specificity of the thioester-containing reactive site of human C3 and its significance to complement activation. Biochem J (1994) 302(Pt 2):429–36. 8092994 10.1042/bj3020429 PMC1137246

[13] Law SK Dodds AW. The internal thioester and the covalent binding properties of the complement proteins C3 and C4. Protein Sci (1997) 6:263–74. 10.1002/pro.5560060201 9041627 PMC2143658

[14] Manderson AP Pickering MC Botto M Walport MJ Parish CR. Continual low-level activation of the classical complement pathway. J Exp Med (2001) 194:747–56. 10.1084/jem.194.6.747 11560991 PMC2195964

[15] Galvan MD Greenlee-Wacker MC Bohlson SS. C1q and phagocytosis: the perfect complement to a good meal. J Leukoc Biol (2012) 92:489–97. 10.1189/jlb.0212099 22715140

[16] Verbovetski I Bychkov H Trahtemberg U Shapira I Hareuveni M Ben-Tal O Opsonization of apoptotic cells by autologous iC3b facilitates clearance by immature dendritic cells, down-regulates DR and CD86, and up-regulates CC chemokine receptor 7. J Exp Med (2002) 196:1553–61. 10.1084/jem.20020263 12486098 PMC2196062

[17] Baudino L Sardini A Ruseva MM Fossati-Jimack L Cook HT Scott D C3 opsonization regulates endocytic handling of apoptotic cells resulting in enhanced T-cell responses to cargo-derived antigens. Proc Natl Acad Sci U S A (2014) 111:1503–8. 10.1073/pnas.1316877111 24474777 PMC3910597

[18] Clarke EV Weist BM Walsh CM Tenner AJ. Complement protein C1q bound to apoptotic cells suppresses human macrophage and dendritic cell-mediated Th17 and Th1 T cell subset proliferation. J Leukoc Biol (2015) 97:147–60. 10.1189/jlb.3A0614-278R 25381385 PMC4377823

[19] Nauta AJ Castellano G Xu W Woltman AM Borrias MC Daha MR Opsonization with C1q and mannose-binding lectin targets apoptotic cells to dendritic cells. J Immunol (2004) 1950(173):3044–50. 10.4049/jimmunol.173.5.3044 15322164

[20] Walport MJ Complement. Second of two parts. N Engl J Med (2001) 344:1140–4. 10.1056/NEJM200104123441506 11297706

[21] Harboe M Mollnes TE. The alternative complement pathway revisited. J Cell Mol Med (2008) 12(4):1074–84. 18419792 10.1111/j.1582-4934.2008.00350.x PMC3865650

[22] Lachmann PJ The amplification loop of the complement pathways. Adv Immunol (2009) 104:115–49. 10.1016/S0065-2776(08)04004-2 20457117

[23] Ghai R Waters P Roumenina LT Gadjeva M Kojouharova MS Reid KBM C1q and its growing family. Immunobiology (2007) 212:253–66. 10.1016/j.imbio.2006.11.001 17544811

[24] Roumenina LT Kantardjiev AA Atanasov BP Waters P Gadjeva M Reid KBM Role of Ca2**+** in the electrostatic stability and the functional activity of the globular domain of human C1q. Biochemistry (2005) 44:14097–109. 10.1021/bi051186n 16245926

[25] Kishore U Ghai R Greenhough TJ Shrive AK Bonifati DM Gadjeva MG Structural and functional anatomy of the globular domain of complement protein C1q. Immunol Lett (2004) 95:113–28. 10.1016/j.imlet.2004.06.015 15388251 PMC3818097

[26] Gadjeva MG Rouseva MM Zlatarova AS Reid KBM Kishore U Kojouharova MS. Interaction of human C1q with IgG and IgM: revisited. Biochemistry (2008) 47:13093–102. 10.1021/bi801131h 19006321

[27] Kojouharova MS Gadjeva MG Tsacheva IG Zlatarova A Roumenina LT Tchorbadjieva MI Mutational analyses of the recombinant globular regions of human C1q A, B, and C chains suggest an essential role for arginine and histidine residues in the C1q-IgG interaction. J Immunol (2004) 1950(172):4351–8. 10.4049/jimmunol.172.7.4351 15034050

[28] Roumenina LT Ruseva MM Zlatarova A Ghai R Kolev M Olova N Interaction of C1q with IgG1, C-reactive protein and pentraxin 3: mutational studies using recombinant globular head modules of human C1q A, B, and C chains. Biochemistry (2006) 45:4093–104. 10.1021/bi052646f 16566583 PMC3874390

[29] Zlatarova AS Rouseva M Roumenina LT Gadjeva M Kolev M Dobrev I Existence of different but overlapping IgG- and IgM-binding sites on the globular domain of human C1q. Biochemistry (2006) 45:9979–88. 10.1021/bi060539v 16906756

[30] Roumenina LT Popov KT Bureeva SV Kojouharova M Gadjeva M Rabheru S Interaction of the globular domain of human C1q with *Salmonella typhimurium* lipopolysaccharide. Biochim Biophys Acta (2008) 1784:1271–6. 10.1016/j.bbapap.2008.04.029 18513495

[31] Albertí S Marqués G Hernández-Allés S Rubires X Tomás JM Vivanco F Interaction between complement subcomponent C1q and the *Klebsiella pneumoniae* porin OmpK36. Infect Immun (1996) 64:4719–25. 8890231 10.1128/iai.64.11.4719-4725.1996 PMC174437

[32] Gaboriaud C Frachet P Thielens NM Arlaud GJ. The human c1q globular domain: structure and recognition of non-immune self ligands. Front Immunol (2011) 2:92. 10.3389/fimmu.2011.00092 22566881 PMC3342031

[33] Nauta AJ Trouw LA Daha MR Tijsma O Nieuwland R Schwaeble WJ Direct binding of C1q to apoptotic cells and cell blebs induces complement activation. Eur J Immunol (2002) 32:1726–36. 10.1002/1521-4141(200206)32:6<1726::AID-IMMU1726>3.0.CO;2-R 12115656

[34] Païdassi H Tacnet-Delorme P Garlatti V Darnault C Ghebrehiwet B Gaboriaud C C1q binds phosphatidylserine and likely acts as a multiligand-bridging molecule in apoptotic cell recognition. J Immunol (2008) 1950(180):2329–38. 10.4049/jimmunol.180.4.2329 18250442 PMC2632962

[35] Païdassi H Tacnet-Delorme P Verneret M Gaboriaud C Houen G Duus K Investigations on the C1q-calreticulin-phosphatidylserine interactions yield new insights into apoptotic cell recognition. J Mol Biol (2011) 408:277–90. 10.1016/j.jmb.2011.02.029 21352829

[36] Païdassi H Tacnet-Delorme P Lunardi T Arlaud GJ Thielens NM Frachet P. The lectin-like activity of human C1q and its implication in DNA and apoptotic cell recognition. FEBS Lett (2008) 582:3111–6. 10.1016/j.febslet.2008.08.001 18703056

[37] Tissot B Daniel R Place C. Interaction of the C1 complex of complement with sulfated polysaccharide and DNA probed by single molecule fluorescence microscopy. Eur J Biochem (2003) 270:4714–20. 10.1046/j.1432-1033.2003.03870.x 14622259

[38] Terrasse R Tacnet-Delorme P Moriscot C Pérard J Schoehn G Vernet T Human and pneumococcal cell surface glyceraldehyde-3-phosphate dehydrogenase (GAPDH) proteins are both ligands of human C1q protein. J Biol Chem (2012) 287:42620–33. 10.1074/jbc.M112.423731 23086952 PMC3522263

[39] Martin M Leffler J Blom AM. Annexin A2 and A5 serve as new ligands for C1q on apoptotic cells. J Biol Chem (2012) 287:33733–44. 10.1074/jbc.M112.341339 22879587 PMC3460470

[40] Ogden CA deCathelineau A Hoffmann PR Bratton D Ghebrehiwet B Fadok VA C1q and mannose binding lectin engagement of cell surface calreticulin and CD91 initiates macropinocytosis and uptake of apoptotic cells. J Exp Med (2001) 194:781–95. 10.1084/jem.194.6.781 11560994 PMC2195958

[41] Steinø A Jørgensen CS Laursen I Houen G. Interaction of C1q with the receptor calreticulin requires a conformational change in C1q. Scand J Immunol (2004) 59:485–95. 10.1111/j.0300-9475.2004.01425.x 15140059

[42] Verneret M Tacnet-Delorme P Osman R Awad R Grichine A Kleman J-P Relative contribution of c1q and apoptotic cell-surface calreticulin to macrophage phagocytosis. J Innate Immun (2014) 6:426–34. 10.1159/000358834 24557008 PMC6741593

[43] Matsushita M Endo Y Taira S Sato Y Fujita T Ichikawa N A novel human serum lectin with collagen- and fibrinogen-like domains that functions as an opsonin. J Biol Chem (1996) 271:2448–54. 10.1074/jbc.271.5.2448 8576206

[44] Bally I Rossi V Lunardi T Thielens NM Gaboriaud C Arlaud GJ. Identification of the C1q-binding sites of human C1r and C1s: a refined three-dimensional model of the C1 complex of complement. J Biol Chem (2009) 284:19340–8. 10.1074/jbc.M109.004473 19473974 PMC2740559

[45] Brier S Pflieger D Le Mignon M Bally I Gaboriaud C Arlaud GJ Mapping surface accessibility of the C1r/C1s tetramer by chemical modification and mass spectrometry provides new insights into assembly of the human C1 complex. J Biol Chem (2010) 285:32251–63. 10.1074/jbc.M110.149112 20592021 PMC2952226

[46] Teillet F Dublet B Andrieu J-P Gaboriaud C Arlaud GJ Thielens NM. The two major oligomeric forms of human mannan-binding lectin: chemical characterization, carbohydrate-binding properties, and interaction with MBL-associated serine proteases. J Immunol (2005) 1950(174):2870–7. 10.4049/jimmunol.174.5.2870 15728497

[47] Thiel S Vorup-Jensen T Stover CM Schwaeble W Laursen SB Poulsen K A second serine protease associated with mannan-binding lectin that activates complement. Nature (1997) 386:506–10. 10.1038/386506a0 9087411

[48] Gaboriaud C Thielens NM Gregory LA Rossi V Fontecilla-Camps JC Arlaud GJ Structure and activation of the C1 complex of complement: unraveling the puzzle. Trends Immunol (2004) 25:368–73. 10.1016/j.it.2004.04.008 15207504

[49] Diebolder CA Beurskens FJ de Jong RN Koning RI Strumane K Lindorfer MA Complement is activated by IgG hexamers assembled at the cell surface. Science (2014) 343:1260–3. 10.1126/science.1248943 24626930 PMC4250092

[50] Cragg MS Morgan SM Chan HTC Morgan BP Filatov AV Johnson PWM Complement-mediated lysis by anti-CD20 mAb correlates with segregation into lipid rafts. Blood (2003) 101:1045–52. 10.1182/blood-2002-06-1761 12393541

[51] Hughes-Jones NC Gorick BD Howard JC Feinstein A. Antibody density on rat red cells determines the rate of activation of the complement component C1. Eur J Immunol (1985) 15:976–80. 10.1002/eji.1830151003 2996908

[52] Preiner J Kodera N Tang J Ebner A Brameshuber M Blaas D IgGs are made for walking on bacterial and viral surfaces. Nat Commun (2014) 5:4394. 10.1038/ncomms5394 25008037

[53] Gaboriaud C Ling WL Thielens NM Bally I Rossi V. Deciphering the fine details of c1 assembly and activation mechanisms: “mission impossible”? Front Immunol (2014) 5:565. 10.3389/fimmu.2014.00565 25414705 PMC4222235

[54] Bally I Ancelet S Moriscot C Gonnet F Mantovani A Daniel R Expression of recombinant human complement C1q allows identification of the C1r/C1s-binding sites. Proc Natl Acad Sci U S A (2013) 110:8650–5. 10.1073/pnas.1304894110 23650384 PMC3666734

[55] Roumenina LT Radanova M Atanasov BP Popov KT Kaveri SV Lacroix-Desmazes S Heme interacts with c1q and inhibits the classical complement pathway. J Biol Chem (2011) 286:16459–69. 10.1074/jbc.M110.206136 21454703 PMC3091251

[56] Arlaud GJ Gaboriaud C Thielens NM Rossi V Bersch B Hernandez JF Structural biology of C1: dissection of a complex molecular machinery. Immunol Rev (2001) 180:136–45. 10.1034/j.1600-065X.2001.1800112.x 11414355

[57] Arlaud GJ Gaboriaud C Garnier G Circolo A Thielens NM Budayova-Spano M Structure, function and molecular genetics of human and murine C1r. Immunobiology (2002) 205:365–82. 10.1078/0171-2985-00139 12396000

[58] Arlaud GJ Gaboriaud C Thielens NM Budayova-Spano M Rossi V Fontecilla-Camps JC. Structural biology of the C1 complex of complement unveils the mechanisms of its activation and proteolytic activity. Mol Immunol (2002) 39:383–94. 10.1016/S0161-5890(02)00143-8 12413689

[59] Budayova-Spano M Grabarse W Thielens NM Hillen H Lacroix M Schmidt M Monomeric structures of the zymogen and active catalytic domain of complement protease c1r: further insights into the c1 activation mechanism. Structure (2002) 1993(10):1509–19. 10.1093/emboj/21.3.231 12429092

[60] Budayova-Spano M Lacroix M Thielens NM Arlaud GJ Fontecilla-Camps JC Gaboriaud C. The crystal structure of the zymogen catalytic domain of complement protease C1r reveals that a disruptive mechanical stress is required to trigger activation of the C1 complex. EMBO J (2002) 21:231–9. 10.1093/emboj/21.3.231 11823416 PMC125823

[61] Gregory LA Thielens NM Arlaud GJ Fontecilla-Camps JC Gaboriaud C. X-ray structure of the Ca2**+**-binding interaction domain of C1s. Insights into the assembly of the C1 complex of complement. J Biol Chem (2003) 278:32157–64. 10.1074/jbc.M305175200 12788922

[62] Girija UV Gingras AR Marshall JE Panchal R Sheikh MA Gál P Structural basis of the C1q/C1s interaction and its central role in assembly of the C1 complex of complement activation. Proc Natl Acad Sci U S A (2013) 110:13916–20. 10.1073/pnas.1311113110 23922389 PMC3752233

[63] Ziccardi RJ Activation of the early components of the classical complement pathway under physiologic conditions. J Immunol (1981) 1950(126):1769–73. 7217665

[64] Kjaer TR Thiel S Andersen GR Toward a structure-based comprehension of the lectin pathway of complement. Mol Immunol (2013) 56:413–22. 10.1016/j.molimm.2013.05.220 23911397

[65] Matsushita M Endo Y Fujita T. Structural and functional overview of the lectin complement pathway: its molecular basis and physiological implication. Arch Immunol Ther Exp (Warsz) (2013) 61:273–83. 10.1007/s00005-013-0229-y 23563865

[66] Frederiksen PD Thiel S Larsen CB Jensenius JC. M-ficolin, an innate immune defence molecule, binds patterns of acetyl groups and activates complement. Scand J Immunol (2005) 62:462–73. 10.1111/j.1365-3083.2005.01685.x 16305643

[67] Krarup A Thiel S Hansen A Fujita T Jensenius JC. L-ficolin is a pattern recognition molecule specific for acetyl groups. J Biol Chem (2004) 279:47513–9. 10.1074/jbc.M407161200 15331601

[68] Héja D Kocsis A Dobó J Szilágyi K Szász R Závodszky P Revised mechanism of complement lectin-pathway activation revealing the role of serine protease MASP-1 as the exclusive activator of MASP-2. Proc Natl Acad Sci U S A (2012) 109:10498–503. 10.1073/pnas.1202588109 22691502 PMC3387078

[69] Dahl MR Thiel S Matsushita M Fujita T Willis AC Christensen T MASP-3 and its association with distinct complexes of the mannan-binding lectin complement activation pathway. Immunity (2001) 15:127–35. 10.1016/S1074-7613(01)00161-3 11485744

[70] Gaboriaud C Gupta RK Martin L Lacroix M Serre L Teillet F The serine protease domain of MASP-3: enzymatic properties and crystal structure in complex with ecotin. PLoS One (2013) 8:e67962. 10.1371/journal.pone.0067962 23861840 PMC3701661

[71] Iwaki D Kanno K Takahashi M Endo Y Lynch NJ Schwaeble WJ Small mannose-binding lectin-associated protein plays a regulatory role in the lectin complement pathway. J Immunol (2006) 1950(177):8626–32. 10.4049/jimmunol.177.12.8626 17142762

[72] Megyeri M Harmat V Major B Végh Á Balczer J Héja D Quantitative characterization of the activation steps of mannan-binding lectin (MBL)-associated serine proteases (MASPs) points to the central role of MASP-1 in the initiation of the complement lectin pathway. J Biol Chem (2013) 288:8922–34. 10.1074/jbc.M112.446500 23386610 PMC3610966

[73] Takahashi M Iwaki D Kanno K Ishida Y Xiong J Matsushita M Mannose-binding lectin (MBL)-associated serine protease (MASP)-1 contributes to activation of the lectin complement pathway. J Immunol (2008) 1950(180):6132–8. 10.4049/jimmunol.180.9.6132 18424734

[74] Yongqing T Drentin N Duncan RC Wijeyewickrema LC Pike RN. Mannose-binding lectin serine proteases and associated proteins of the lectin pathway of complement: two genes, five proteins and many functions? Biochim Biophys Acta (2012) 1824:253–62. 10.1016/j.bbapap.2011.05.021 21664989

[75] Gál P Harmat V Kocsis A Bián T Barna L Ambrus G A true autoactivating enzyme. Structural insight into mannose-binding lectin-associated serine protease-2 activations. J Biol Chem (2005) 280:33435–44. 10.1074/jbc.M506051200 16040602

[76] Degn SE Kjaer TR Kidmose RT Jensen L Hansen AG Tekin M Complement activation by ligand-driven juxtaposition of discrete pattern recognition complexes. Proc Natl Acad Sci U S A (2014) 111:13445–50. 10.1073/pnas.1406849111 25197071 PMC4169954

[77] Kidmose RT Laursen NS Dobó J Kjaer TR Sirotkina S Yatime L Structural basis for activation of the complement system by component C4 cleavage. Proc Natl Acad Sci U S A (2012) 109:15425–30. 10.1073/pnas.1208031109 22949645 PMC3458355

[78] Beinrohr L Dobó J Závodszky P Gál P. C1, MBL-MASPs and C1-inhibitor: novel approaches for targeting complement-mediated inflammation. Trends Mol Med (2008) 14:511–21. 10.1016/j.molmed.2008.09.009 18977695

[79] Davis AE Lu F Mejia P. C1 inhibitor, a multi-functional serine protease inhibitor. Thromb Haemost (2010) 104:886–93. 10.1160/TH10-01-0073 20806108

[80] Ziccardi RJ A new role for C-1-inhibitor in homeostasis: control of activation of the first component of human complement. J Immunol (1982) 1950(128):2505–8. 7077078

[81] Longhurst H Cicardi M. Hereditary angio-oedema. Lancet (2012) 379:474–81. 10.1016/S0140-6736(11)60935-5 22305226

[82] Galanakis DK Ghebrehiwet B. A unique property of a plasma proteoglycan, the C1q inhibitor. An anticoagulant state resulting from its binding to fibrinogen. J Clin Invest (1994) 93:303–10. 10.1172/JCI116960 8282801 PMC293766

[83] Roumenina L Bureeva S Kantardjiev A Karlinsky D Andia-Pravdivy JE Sim R Complement C1q-target proteins recognition is inhibited by electric moment effectors. J Mol Recognit (2007) 20:405–15. 10.1002/jmr.853 17929239

[84] Ferreira V Valck C Sánchez G Gingras A Tzima S Molina MC The classical activation pathway of the human complement system is specifically inhibited by calreticulin from *Trypanosoma cruzi*. J Immunol (2004) 1950(172):3042–50. 10.4049/jimmunol.172.5.3042 14978109

[85] Yadav S Gupta S Selvaraj C Doharey PK Verma A Singh SK In silico and in vitro studies on the protein-protein interactions between *Brugia malayi* immunomodulatory protein calreticulin and human C1q. PLoS One (2014) 9:e106413. 10.1371/journal.pone.0106413 25184227 PMC4153637

[86] Degn SE Hansen AG Steffensen R Jacobsen C Jensenius JC Thiel S. MAp44, a human protein associated with pattern recognition molecules of the complement system and regulating the lectin pathway of complement activation. J Immunol (2009) 1950(183):7371–8. 10.4049/jimmunol.0902388 19917686

[87] Paréj K Dobó J Závodszky P Gál P. The control of the complement lectin pathway activation revisited: both C1-inhibitor and antithrombin are likely physiological inhibitors, while **α**2-macroglobulin is not. Mol Immunol (2013) 54:415–22. 10.1016/j.molimm.2013.01.009 23399388

[88] Presanis JS Hajela K Ambrus G Gál P Sim RB. Differential substrate and inhibitor profiles for human MASP-1 and MASP-2. Mol Immunol (2004) 40:921–9. 10.1016/j.molimm.2003.10.013 14725788

[89] Hamad OA Nilsson PH Wouters D Lambris JD Ekdahl KN Nilsson B. Complement component C3 binds to activated normal platelets without preceding proteolytic activation and promotes binding to complement receptor 1. J Immunol (2010) 1950(184):2686–92. 10.4049/jimmunol.0902810 20139276 PMC2953618

[90] Saggu G Cortes C Emch HN Ramirez G Worth RG Ferreira VP. Identification of a novel mode of complement activation on stimulated platelets mediated by properdin and C3(H2O). J Immunol (2013) 190:6457–67. 10.4049/jimmunol.1300610 23677468 PMC3784323

[91] Fearon DT Austen KF. Properdin: binding to C3b and stabilization of the C3b-dependent C3 convertase. J Exp Med (1975) 142:856–63. 10.1084/jem.142.4.856 1185108 PMC2189935

[92] Hourcade DE. The role of properdin in the assembly of the alternative pathway C3 convertases of complement. J Biol Chem (2006) 281:2128–32. 10.1074/jbc.M508928200 16301317

[93] Kemper C Atkinson JP Hourcade DE. Properdin: emerging roles of a pattern-recognition molecule. Annu Rev Immunol (2010) 28:131–55. 10.1146/annurev-immunol-030409-101250 19947883

[94] Zaferani A Vivès RR van der Pol P Hakvoort JJ Navis GJ van Goor H Identification of tubular heparan sulfate as a docking platform for the alternative complement component properdin in proteinuric renal disease. J Biol Chem (2011) 286:5359–67. 10.1074/jbc.M110.167825 21135110 PMC3037648

[95] Camous L Roumenina L Bigot S Brachemi S Frémeaux-Bacchi V Lesavre P Complement alternative pathway acts as a positive feedback amplification of neutrophil activation. Blood (2011) 117:1340–9. 10.1182/blood-2010-05-283564 21063021

[96] O’Flynn J Dixon KO Faber Krol MC Daha MR van Kooten C. Myeloperoxidase directs properdin-mediated complement activation. J Innate Immun (2014) 6:417–25. 10.1159/000356980 24355864 PMC6741500

[97] Goligorsky MS Patschan D Kuo M-C. Weibel–Palade bodies – sentinels of acute stress. Nat Rev Nephrol (2009) 5:423–6. 10.1038/nrneph.2009.87 19556996

[98] del Conde I. Platelet activation leads to activation and propagation of the complement system. J Exp Med (2005) 201:871–9. 10.1084/jem.20041497 15781579 PMC2213112

[99] Morigi M Galbusera M Gastoldi S Locatelli M Buelli S Pezzotta A Alternative pathway activation of complement by Shiga toxin promotes exuberant C3a formation that triggers microvascular thrombosis. J Immunol (2011) 187:172–80. 10.4049/jimmunol.1100491 21642543

[100] Evans KJ Hansen DS van Rooijen N Buckingham LA Schofield L. Severe malarial anemia of low parasite burden in rodent models results from accelerated clearance of uninfected erythrocytes. Blood (2006) 107:1192–9. 10.1182/blood-2005-08-3460 16210332 PMC1895912

[101] Pawluczkowycz AW Lindorfer MA Waitumbi JN Taylor RP. Hematin promotes complement alternative pathway-mediated deposition of C3 activation fragments on human erythrocytes: potential implications for the pathogenesis of anemia in malaria. J Immunol (2007) 1950(179):5543–52. 10.4049/jimmunol.179.8.5543 17911641

[102] Frimat M Tabarin F Dimitrov JD Poitou C Halbwachs-Mecarelli L Fremeaux-Bacchi V Complement activation by heme as a secondary hit for atypical hemolytic uremic syndrome. Blood (2013) 122:282–92. 10.1182/blood-2013-03-489245 23692858

[103] Belcher JD Chen C Nguyen J Milbauer L Abdulla F Alayash AI Heme triggers TLR4 signaling leading to endothelial cell activation and vaso-occlusion in murine sickle cell disease. Blood (2014) 123:377–90. 10.1182/blood-2013-04-495887 24277079 PMC3894494

[104] Forneris F Ricklin D Wu J Tzekou A Wallace RS Lambris JD Structures of C3b in complex with factors B and D give insight into complement convertase formation. Science (2010) 330:1816–20. 10.1126/science.1195821 21205667 PMC3087196

[105] Janssen BJC Gomes L Koning RI Svergun DI Koster AJ Fritzinger DC Insights into complement convertase formation based on the structure of the factor B-cobra venom factor complex. EMBO J (2009) 28:2469–78. 10.1038/emboj.2009.184 19574954 PMC2735180

[106] Torreira E Tortajada A Montes T Rodríguez de Córdoba S Llorca O. Coexistence of closed and open conformations of complement factor B in the alternative pathway C3bB(Mg2**+**) proconvertase. J Immunol (2009) 1950(183):7347–51. 10.4049/jimmunol.0902310 19890040

[107] Yamauchi Y Stevens JW Macon KJ Volanakis JE. Recombinant and native zymogen forms of human complement factor D. J Immunol (1994) 1950(152):3645–53. 8144940

[108] Iwaki D Kanno K Takahashi M Endo Y Matsushita M Fujita T. The role of mannose-binding lectin-associated serine protease-3 in activation of the alternative complement pathway. J Immunol (2011) 1950(187):3751–8. 10.4049/jimmunol.1100280 21865552

[109] Takahashi M Ishida Y Iwaki D Kanno K Suzuki T Endo Y Essential role of mannose-binding lectin-associated serine protease-1 in activation of the complement factor D. J Exp Med (2010) 207:29–37. 10.1084/jem.20090633 20038603 PMC2812541

[110] Ruseva MM Takahashi M Fujita T Pickering MC. C3 dysregulation due to factor H deficiency is mannan-binding lectin-associated serine proteases (MASP)-1 and MASP-3 independent in vivo. Clin Exp Immunol (2014) 176:84–92. 10.1111/cei.12244 24279761 PMC3958157

[111] Degn SE Jensen L Hansen AG Duman D Tekin M Jensenius JC Mannan-binding lectin-associated serine protease (MASP)-1 is crucial for lectin pathway activation in human serum, whereas neither MASP-1 nor MASP-3 is required for alternative pathway function. J Immunol (2012) 1950(189):3957–69. 10.4049/jimmunol.1201736 22966085

[112] Milder FJ Gomes L Schouten A Janssen BJC Huizinga EG Romijn RA Factor B structure provides insights into activation of the central protease of the complement system. Nat Struct Mol Biol (2007) 14:224–8. 10.1038/nsmb1210 17310251

[113] Rooijakkers SHM Wu J Ruyken M van Domselaar R Planken KL Tzekou A Structural and functional implications of the alternative complement pathway C3 convertase stabilized by a staphylococcal inhibitor. Nat Immunol (2009) 10:721–7. 10.1038/ni.1756 19503103 PMC2729104

[114] Bexborn F Andersson PO Chen H Nilsson B Ekdahl KN. The tick-over theory revisited: formation and regulation of the soluble alternative complement C3 convertase (C3(H2O)Bb). Mol Immunol (2008) 45:2370–9. 10.1016/j.molimm.2007.11.003 18096230 PMC2701500

[115] Krishnan V Xu Y Macon K Volanakis JE Narayana SVL. The structure of C2b, a fragment of complement component C2 produced during C3 convertase formation. Acta Crystallogr D Biol Crystallogr (2009) 65:266–74. 10.1107/S0907444909000389 19237749 PMC2651757

[116] Smith M Kerr M Cleavage of the second component of complement by plasma proteases: implications in hereditary C1-inhibitor deficiency. Immunol (1985) 56:561–70. PMC1453753 2934317

[117] Aplan A Bradykinin and the pathogenesis of hereditary angioedema. World Allergy Organ J. (2011) 4(4):73–5. 23282402 10.1097/WOX.0b013e318216b7b2 PMC3651110

[118] Gros P Milder FJ Janssen BJC Complement driven by conformational changes. Nat Rev Immunol (2008) 8:48–58. 10.1038/nri2231 18064050

[119] Pangburn MK Müller-Eberhard HJ. The C3 convertase of the alternative pathway of human complement. Enzymic properties of the bimolecular proteinase. Biochem J (1986) 235:723–30. 3638964 10.1042/bj2350723 PMC1146747

[120] Kouser L Abdul-Aziz M Nayak A Stover CM Sim RB Kishore U. Properdin and factor h: opposing players on the alternative complement pathway “see-saw”. Front Immunol (2013) 4:93. 10.3389/fimmu.2013.00093 23630525 PMC3632793

[121] Schwaeble W Dippold WG Schäfer MK Pohla H Jonas D Luttig B Properdin, a positive regulator of complement activation, is expressed in human T cell lines and peripheral blood T cells. J Immunol (1993) 1950(151):2521–8. 8360474

[122] Schwaeble W Huemer HP Möst J Dierich MP Ströbel M Claus C Expression of properdin in human monocytes. Eur J Biochem (1994) 219:759–64. 10.1111/j.1432-1033.1994.tb18555.x 8112326

[123] Wirthmueller U Dewald B Thelen M Schäfer MK Stover C Whaley K Properdin, a positive regulator of complement activation, is released from secondary granules of stimulated peripheral blood neutrophils. J Immunol (1997) 1950(158):4444–51. 9127010

[124] Stover CM Luckett JC Echtenacher B Dupont A Figgitt SE Brown J Properdin plays a protective role in polymicrobial septic peritonitis. J Immunol (2008) 1950(180):3313–8. 10.4049/jimmunol.180.5.3313 18292556

[125] Sun Z Almogren A Furtado PB Chowdhury B Kerr MA Perkins SJ. Semi-extended solution structure of human myeloma immunoglobulin D determined by constrained X-ray scattering. J Mol Biol (2005) 353:155–73. 10.1016/j.jmb.2005.07.072 16157351

[126] Pangburn MK. Analysis of the natural polymeric forms of human properdin and their functions in complement activation. J Immunol (1989) 1950(142):202–7. 2909614

[127] Cortes C Ohtola JA Saggu G Ferreira VP. Local release of properdin in the cellular microenvironment: role in pattern recognition and amplification of the alternative pathway of complement. Front Immunol (2012) 3:412. 10.3389/fimmu.2012.00412 23335922 PMC3547370

[128] Ferreira VP Cortes C Pangburn MK. Native polymeric forms of properdin selectively bind to targets and promote activation of the alternative pathway of complement. Immunobiology (2010) 215:932–40. 10.1016/j.imbio.2010.02.002 20382442 PMC2949450

[129] Alcorlo M Tortajada A Rodríguez de Córdoba S Llorca O. Structural basis for the stabilization of the complement alternative pathway C3 convertase by properdin. Proc Natl Acad Sci U S A (2013) 110:13504–9. 10.1073/pnas.1309618110 23901101 PMC3746899

[130] Kajander T Lehtinen MJ Hyvärinen S Bhattacharjee A Leung E Isenman DE Dual interaction of factor H with C3d and glycosaminoglycans in host-nonhost discrimination by complement. Proc Natl Acad Sci U S A (2011) 108:2897–902. 10.1073/pnas.1017087108 21285368 PMC3041134

[131] Morgan HP Schmidt CQ Guariento M Blaum BS Gillespie D Herbert AP Structural basis for engagement by complement factor H of C3b on a self surface. Nat Struct Mol Biol (2011) 18:463–70. 10.1038/nsmb.2018 21317894 PMC3512577

[132] Wu J Wu Y-Q Ricklin D Janssen BJC Lambris JD Gros P. Structure of C3b-factor H and implications for host protection by complement regulators. Nat Immunol (2009) 10:728–33. 10.1038/ni.1755 19503104 PMC2713992

[133] Lambris JD Lao Z Oglesby TJ Atkinson JP Hack CE Becherer JD. Dissection of CR1, factor H, membrane cofactor protein, and factor B binding and functional sites in the third complement component. J Immunol (1996) 1950(156):4821–32. 8648130

[134] Roversi P Johnson S Caesar JJE McLean F Leath KJ Tsiftsoglou SA Structural basis for complement factor I control and its disease-associated sequence polymorphisms. Proc Natl Acad Sci U S A (2011) 108:12839–44. 10.1073/pnas.1102167108 21768352 PMC3150940

[135] Davis AE Harrison RA Lachmann PJ. Physiologic inactivation of fluid phase C3b: isolation and structural analysis of C3c, C3d,g (alpha 2D), and C3g. J Immunol (1984) 1950(132):1960–6. 6607952

[136] Andrews PW Knowles BB Parkar M Pym B Stanley K Goodfellow PN. A human cell-surface antigen defined by a monoclonal antibody and controlled by a gene on human chromosome 1. Ann Hum Genet (1985) 49:31–9. 10.1111/j.1469-1809.1985.tb01673.x 2416262

[137] Liszewski MK Leung MK Atkinson JP. Membrane cofactor protein: importance of N- and O-glycosylation for complement regulatory function. J Immunol (1998) 1950(161):3711–8. 9759896

[138] Barilla-LaBarca ML Liszewski MK Lambris JD Hourcade D Atkinson JP. Role of membrane cofactor protein (CD46) in regulation of C4b and C3b deposited on cells. J Immunol (2002) 1950(168):6298–304. 10.4049/jimmunol.168.12.6298 12055245

[139] Liszewski MK Post TW Atkinson JP. Membrane cofactor protein (MCP or CD46): newest member of the regulators of complement activation gene cluster. Annu Rev Immunol (1991) 9:431–55. 10.1146/annurev.iy.09.040191.002243 1910685

[140] Persson BD Schmitz NB Santiago C Zocher G Larvie M Scheu U Structure of the extracellular portion of CD46 provides insights into its interactions with complement proteins and pathogens. PLoS Pathog (2010) 6:e1001122. 10.1371/journal.ppat.1001122 20941397 PMC2947992

[141] Liszewski MK Leung M Cui W Subramanian VB Parkinson J Barlow PN Dissecting sites important for complement regulatory activity in membrane cofactor protein (MCP; CD46). J Biol Chem (2000) 275:37692–701. 10.1074/jbc.M004650200 10960475

[142] Schramm EC Roumenina LT Rybkine T Chauvet S Vieira-Martins P Hue C Functional mapping of the interactions between complement C3 and regulatory proteins using atypical hemolytic uremic syndrome-associated mutations. Blood (2015). 125(15):2359–69. 10.1182/blood-2014-10-609073 25608561 PMC4392009

[143] Klickstein LB Bartow TJ Miletic V Rabson LD Smith JA Fearon DT. Identification of distinct C3b and C4b recognition sites in the human C3b/C4b receptor (CR1, CD35) by deletion mutagenesis. J Exp Med (1988) 168:1699–717. 10.1084/jem.168.5.1699 2972794 PMC2189104

[144] Wong WW Cahill JM Rosen MD Kennedy CA Bonaccio ET Morris MJ Structure of the human CR1 gene. Molecular basis of the structural and quantitative polymorphisms and identification of a new CR1-like allele. J Exp Med (1989) 169:847–63. 10.1084/jem.169.3.847 2564414 PMC2189269

[145] Krych M Hauhart R Atkinson JP. Structure-function analysis of the active sites of complement receptor type 1. J Biol Chem (1998) 273:8623–9. 10.1074/jbc.273.15.8623 9535836

[146] Smith BO Mallin RL Krych-Goldberg M Wang X Hauhart RE Bromek K Structure of the C3b binding site of CR1 (CD35), the immune adherence receptor. Cell (2002) 108:769–80. 10.1016/S0092-8674(02)00672-4 11955431

[147] Oran AE Isenman DE. Identification of residues within the 727-767 segment of human complement component C3 important for its interaction with factor H and with complement receptor 1 (CR1, CD35). J Biol Chem (1999) 274:5120–30. 10.1074/jbc.274.8.5120 9988761

[148] Blom AM Webb J Villoutreix BO Dahlbäck B. A cluster of positively charged amino acids in the C4BP alpha-chain is crucial for C4b binding and factor I cofactor function. J Biol Chem (1999) 274:19237–45. 10.1074/jbc.274.27.19237 10383431

[149] Blom AM Kask L Dahlbäck B. Structural requirements for the complement regulatory activities of C4BP. J Biol Chem (2001) 276:27136–44. 10.1074/jbc.M102445200 11369776

[150] Blom AM Villoutreix BO Dahlbäck B. Mutations in alpha-chain of C4BP that selectively affect its factor I cofactor function. J Biol Chem (2003) 278:43437–42. 10.1074/jbc.M306620200 12893820

[151] Rawal N Rajagopalan R Salvi VP. Stringent regulation of complement lectin pathway C3/C5 convertase by C4b-binding protein (C4BP). Mol Immunol (2009) 46:2902–10. 10.1016/j.molimm.2009.07.006 19660812 PMC2770428

[152] Hofmeyer T Schmelz S Degiacomi MT Dal Peraro M Daneschdar M Scrima A Arranged sevenfold: structural insights into the C-terminal oligomerization domain of human C4b-binding protein. J Mol Biol (2013) 425:1302–17. 10.1016/j.jmb.2012.12.017 23274142

[153] Kask L Hillarp A Ramesh B Dahlbäck B Blom AM. Structural requirements for the intracellular subunit polymerization of the complement inhibitor C4b-binding protein. Biochemistry (2002) 41:9349–57. 10.1021/bi025980+ 12135356

[154] Ziccardi RJ Dahlback B Müller-Eberhard HJ. Characterization of the interaction of human C4b-binding protein with physiological ligands. J Biol Chem (1984) 259:13674–9. 6334079

[155] Dahlbäck B Smith CA Müller-Eberhard HJ. Visualization of human C4b-binding protein and its complexes with vitamin K-dependent protein S and complement protein C4b. Proc Natl Acad Sci U S A (1983) 80:3461–5. 10.1073/pnas.80.11.3461 6222381 PMC394064

[156] Delvaeye M Noris M De Vriese A Esmon CT Esmon NL Ferrell G Thrombomodulin mutations in atypical hemolytic-uremic syndrome. N Engl J Med (2009) 361:345–57. 10.1056/NEJMoa0810739 19625716 PMC3530919

[157] Rayes J Roumenina LT Dimitrov JD Repessé Y Ing M Christophe O The interaction between factor H and VWF increases factor H cofactor activity and regulates VWF prothrombotic status. Blood (2014) 123:121–5. 10.1182/blood-2013-04-495853 24014239

[158] Feng S Liang X Kroll MH Chung DW Afshar-Kharghan V. Von Willebrand Factor is a cofactor in complement regulation. Blood (2014) 125(6):1034–7. 10.1182/blood-2014-06-585430 25395424 PMC4319234

[159] Krych-Goldberg M Hauhart RE Subramanian VB Yurcisin BM Crimmins DL Hourcade DE Decay accelerating activity of complement receptor type 1 (CD35). Two active sites are required for dissociating C5 convertases. J Biol Chem (1999) 274:31160–8. 10.1074/jbc.274.44.31160 10531307

[160] Nicholson-Weller A Wang CE. Structure and function of decay accelerating factor CD55. J Lab Clin Med (1994) 123:485–91. 7511675

[161] Hourcade DE Mitchell L Kuttner-Kondo LA Atkinson JP Medof ME. Decay-accelerating factor (DAF), complement receptor 1 (CR1), and factor H dissociate the complement AP C3 convertase (C3bBb) via sites on the type A domain of Bb. J Biol Chem (2002) 277:1107–12. 10.1074/jbc.M109322200 11694537

[162] Harris CL Abbott RJM Smith RA Morgan BP Lea SM. Molecular dissection of interactions between components of the alternative pathway of complement and decay accelerating factor (CD55). J Biol Chem (2005) 280:2569–78. 10.1074/jbc.M410179200 15536079

[163] Harris CL Pettigrew DM Lea SM Morgan BP. Decay-accelerating factor must bind both components of the complement alternative pathway C3 convertase to mediate efficient decay. J Immunol (2007) 178:352–9. 10.4049/jimmunol.178.1.352 17182573

[164] Hasan RJ Pawelczyk E Urvil PT Venkatarajan MS Goluszko P Kur J Structure-function analysis of decay-accelerating factor: identification of residues important for binding of the *Escherichia coli* Dr adhesin and complement regulation. Infect Immun (2002) 70:4485–93. 10.1128/IAI.70.8.4485-4493.2002 12117960 PMC128121

[165] Kazatchkine MD Fearon DT Austen KF Human alternative complement pathway: membrane-associated sialic acid regulates the competition between B and beta1 H for cell-bound C3b. J Immunol (1979) 1950(122):75–81. 762425

[166] Weiler JM Daha MR Austen KF Fearon DT. Control of the amplification convertase of complement by the plasma protein beta1H. Proc Natl Acad Sci U S A (1976) 73:3268–72. 10.1073/pnas.73.9.3268 1067618 PMC431003

[167] Whaley K Ruddy S. Modulation of the alternative complement pathways by beta 1 H globulin. J Exp Med (1976) 144:1147–63. 10.1084/jem.144.5.1147 62817 PMC2190448

[168] Perkins SJ Nan R Li K Khan S Miller A. Complement factor H-ligand interactions: self-association, multivalency and dissociation constants. Immunobiology (2012) 217:281–97. 10.1016/j.imbio.2011.10.003 22137027

[169] Zipfel PF Skerka C. FHL-1/reconectin: a human complement and immune regulator with cell-adhesive function. Immunol Today (1999) 20:135–40. 10.1016/S0167-5699(98)01432-7 10203705

[170] Hellwage J Jokiranta TS Friese MA Wolk TU Kampen E Zipfel PF Complement C3b/C3d and cell surface polyanions are recognized by overlapping binding sites on the most carboxyl-terminal domain of complement factor H. J Immunol (2002) 1950(169):6935–44. 10.4049/jimmunol.169.12.6935 12471127

[171] Jokiranta TS Hellwage J Koistinen V Zipfel PF Meri S. Each of the three binding sites on complement factor H interacts with a distinct site on C3b. J Biol Chem (2000) 275:27657–62. 10.1074/jbc.M002903200 10837479

[172] Morgan HP Mertens HDT Guariento M Schmidt CQ Soares DC Svergun DI Structural analysis of the C-terminal region (modules 18–20) of complement regulator factor H (FH). PLoS One (2012) 7(2):e32187. 10.1371/journal.pone.0032187 22389686 PMC3289644

[173] Bhattacharjee A Lehtinen MJ Kajander T Goldman A Jokiranta TS. Both domain 19 and domain 20 of factor H are involved in binding to complement C3b and C3d. Mol Immunol (2010) 47:1686–91. 10.1016/j.molimm.2010.03.007 20378178

[174] Blaum BS Hannan JP Herbert AP Kavanagh D Uhrín D Stehle T. Structural basis for sialic acid-mediated self-recognition by complement factor H. Nat Chem Biol (2014) 11(1):77–82. 10.1038/nchembio.1696 25402769

[175] Ferreira VP Herbert AP Cortés C McKee KA Blaum BS Esswein ST The binding of factor H to a complex of physiological polyanions and C3b on cells is impaired in atypical hemolytic uremic syndrome. J Immunol (2009) 1950(182):7009–18. 10.4049/jimmunol.0804031 19454698 PMC2696619

[176] Jokiranta TS Jaakola V-P Lehtinen MJ Pärepalo M Meri S Goldman A. Structure of complement factor H carboxyl-terminus reveals molecular basis of atypical haemolytic uremic syndrome. EMBO J (2006) 25:1784–94. 10.1038/sj.emboj.7601052 16601698 PMC1440827

[177] Lehtinen MJ Rops AL Isenman DE van der Vlag J Jokiranta TS. Mutations of factor H impair regulation of surface-bound C3b by three mechanisms in atypical hemolytic uremic syndrome. J Biol Chem (2009) 284:15650–8. 10.1074/jbc.M900814200 19351878 PMC2708861

[178] Okemefuna AI Li K Nan R Ormsby RJ Sadlon T Gordon DL Multimeric interactions between complement factor H and its C3d ligand provide new insight on complement regulation. J Mol Biol (2009) 391:119–35. 10.1016/j.jmb.2009.06.013 19505474

[179] Oppermann M Manuelian T Józsi M Brandt E Jokiranta TS Heinen S The C-terminus of complement regulator factor H mediates target recognition: evidence for a compact conformation of the native protein. Clin Exp Immunol (2006) 144:342–52. 10.1111/j.1365-2249.2006.03071.x 16634809 PMC1809651

[180] Prosser BE Johnson S Roversi P Herbert AP Blaum BS Tyrrell J Structural basis for complement factor H linked age-related macular degeneration. J Exp Med (2007) 204:2277–83. 10.1084/jem.20071069 17893204 PMC2118454

[181] Schmidt CQ Herbert AP Mertens HDT Guariento M Soares DC Uhrin D The central portion of factor H (modules 10-15) is compact and contains a structurally deviant CCP module. J Mol Biol (2010) 395:105–22. 10.1016/j.jmb.2009.10.010 19835885 PMC2806952

[182] Makou E Mertens HDT Maciejewski M Soares DC Matis I Schmidt CQ Solution structure of CCP modules 10-12 illuminates functional architecture of the complement regulator, factor H. J Mol Biol (2012) 424:295–312. 10.1016/j.jmb.2012.09.013 23017427 PMC4068365

[183] Schmidt CQ Herbert AP Kavanagh D Gandy C Fenton CJ Blaum BS A new map of glycosaminoglycan and C3b binding sites on factor H. J Immunol (2008) 1950(181):2610–9. 10.4049/jimmunol.181.4.2610 18684951

[184] Sharma AK Pangburn MK. Identification of three physically and functionally distinct binding sites for C3b in human complement factor H by deletion mutagenesis. Proc Natl Acad Sci U S A (1996) 93:10996–1001. 10.1073/pnas.93.20.10996 8855297 PMC38272

[185] Blackmore TK Hellwage J Sadlon TA Higgs N Zipfel PF Ward HM Identification of the second heparin-binding domain in human complement factor H. J Immunol (1998) 1950(160):3342–8. 9531293

[186] Clark SJ Bishop PN Day AJ The proteoglycan glycomatrix: a sugar microenvironment essential for complement regulation. Inflammation (2013) 4:412 10.3389/fimmu.2013.00412 PMC3840399 24324472

[187] Herbert AP Deakin JA Schmidt CQ Blaum BS Egan C Ferreira VP Structure shows that a glycosaminoglycan and protein recognition site in factor H is perturbed by age-related macular degeneration-linked single nucleotide polymorphism. J Biol Chem (2007) 282:18960–8. 10.1074/jbc.M609636200 17360715

[188] Ormsby RJ Jokiranta TS Duthy TG Griggs KM Sadlon TA Giannakis E Localization of the third heparin-binding site in the human complement regulator factor H1. Mol Immunol (2006) 43:1624–32. 10.1016/j.molimm.2005.09.012 16263173

[189] Perkins SJ Fung KW Khan S. Molecular interactions between complement factor H and its heparin and heparan sulfate ligands. Front Immunol (2014) 5:126. 10.3389/fimmu.2014.00126 24744754 PMC3978290

[190] Zaferani A Vivès RR van der Pol P Navis GJ Daha MR van Kooten C Factor h and properdin recognize different epitopes on renal tubular epithelial heparan sulfate. J Biol Chem (2012) 287:31471–81. 10.1074/jbc.M112.380386 22815489 PMC3438980

[191] Roumenina LT Roquigny R Blanc C Poulain N Ngo S Dragon-Durey M-A Functional evaluation of factor H genetic and acquired abnormalities: application for atypical hemolytic uremic syndrome (aHUS). Methods Mol Biol (2014) 1100:237–47. 10.1007/978-1-62703-724-2_19 24218264

[192] Clark SJ Ridge LA Herbert AP Hakobyan S Mulloy B Lennon R Tissue-specific host recognition by complement factor H is mediated by differential activities of its glycosaminoglycan-binding regions. J Immunol (2013) 1950(190):2049–57. 10.4049/jimmunol.1201751 23365078 PMC3672945

[193] Langford-Smith A Day AJ Bishop PN Clark SJ. Complementing the sugar code: role of GAGs and sialic acid in complement regulation. Mol Innate Immun (2015) 6:25. 10.3389/fimmu.2015.00025 25699044 PMC4313701

[194] Edwards AO Ritter R Abel KJ Manning A Panhuysen C Farrer LA. Complement factor H polymorphism and age-related macular degeneration. Science (2005) 308:421–4. 10.1126/science.1110189 15761121

[195] Hageman GS Anderson DH Johnson LV Hancox LS Taiber AJ Hardisty LI A common haplotype in the complement regulatory gene factor H (HF1/CFH) predisposes individuals to age-related macular degeneration. Proc Natl Acad Sci U S A (2005) 102:7227–32. 10.1073/pnas.0501536102 15870199 PMC1088171

[196] Haines JL Hauser MA Schmidt S Scott WK Olson LM Gallins P Complement factor H variant increases the risk of age-related macular degeneration. Science (2005) 308:419–21. 10.1126/science.1110359 15761120

[197] Klein RJ Zeiss C Chew EY Tsai J-Y Sackler RS Haynes C Complement factor H polymorphism in age-related macular degeneration. Science (2005) 308:385–9. 10.1126/science.1109557 15761122 PMC1512523

[198] Shaw PX Zhang L Zhang M Du H Zhao L Lee C Complement factor H genotypes impact risk of age-related macular degeneration by interaction with oxidized phospholipids. Proc Natl Acad Sci U S A (2012) 109:13757–62. 10.1073/pnas.1121309109 22875704 PMC3427125

[199] Weismann D Binder CJ. The innate immune response to products of phospholipid peroxidation. Biochim Biophys Acta (2012) 1818:2465–75. 10.1016/j.bbamem.2012.01.018 22305963 PMC3790971

[200] Weismann D Hartvigsen K Lauer N Bennett KL Scholl HPN Charbel Issa P Complement factor H binds malondialdehyde epitopes and protects from oxidative stress. Nature (2011) 478:76–81. 10.1038/nature10449 21979047 PMC4826616

[201] Okemefuna AI Nan R Miller A Gor J Perkins SJ. Complement factor H binds at two independent sites to C-reactive protein in acute phase concentrations. J Biol Chem (2010) 285:1053–65. 10.1074/jbc.M109.044529 19850925 PMC2801232

[202] Skerka C Chen Q Fremeaux-Bacchi V Roumenina LT Complement factor H related proteins (CFHRs). Mol Immunol (2013) 56:170–80. 10.1016/j.molimm.2013.06.001 23830046

[203] Goicoechea de Jorge E Caesar JJE Malik TH Patel M Colledge M Johnson S Dimerization of complement factor H-related proteins modulates complement activation in vivo. Proc Natl Acad Sci U S A (2013) 110:4685–90. 10.1073/pnas.1219260110 23487775 PMC3606973

[204] Tortajada A Yébenes H Abarrategui-Garrido C Anter J García-Fernández JM Martínez-Barricarte R C3 glomerulopathy-associated CFHR1 mutation alters FHR oligomerization and complement regulation. J Clin Invest (2013) 123:2434–46. 10.1172/JCI68280 23728178 PMC3668852

[205] Fritsche LG Lauer N Hartmann A Stippa S Keilhauer CN Oppermann M An imbalance of human complement regulatory proteins CFHR1, CFHR3 and factor H influences risk for age-related macular degeneration (AMD). Hum Mol Genet (2010) 19:4694–704. 10.1093/hmg/ddq399 20843825

[206] Heinen S Hartmann A Lauer N Wiehl U Dahse H-M Schirmer S Factor H-related protein 1 (CFHR-1) inhibits complement C5 convertase activity and terminal complex formation. Blood (2009) 114:2439–47. 10.1182/blood-2009-02-205641 19528535

[207] Bubeck D. The making of a macromolecular machine: assembly of the membrane attack complex. Biochemistry (2014) 53:1908–15. 10.1021/bi500157z 24597946

[208] Kinoshita T Takata Y Kozono H Takeda J Hong KS Inoue K. C5 convertase of the alternative complement pathway: covalent linkage between two C3b molecules within the trimolecular complex enzyme. J Immunol (1988) 1950(141):3895–901. 3183384

[209] Kim YU Carroll MC Isenman DE Nonaka M Pramoonjago P Takeda J Covalent binding of C3b to C4b within the classical complement pathway C5 convertase. Determination of amino acid residues involved in ester linkage formation. J Biol Chem (1992) 267:4171–6. 1740458

[210] Barrio E Antón LC Marqués G Sánchez A Vivanco F. Formation of covalently linked C3-C3 dimers on IgG immune aggregates. Eur J Immunol (1991) 21:343–9. 10.1002/eji.1830210215 1999223

[211] Laursen NS Andersen KR Braren I Spillner E Sottrup-Jensen L Andersen GR. Substrate recognition by complement convertases revealed in the C5-cobra venom factor complex. EMBO J (2011) 30:606–16. 10.1038/emboj.2010.341 21217642 PMC3034014

[212] Mortensen S Kidmose RT Petersen SV Szilágyi Á Prohászka Z Andersen GR Structural Basis for the Function of Complement Component C4 within the Classical and Lectin Pathways of Complement. J Immunol (2015) 194:5488–96. 10.4049/jimmunol.1500087 25911760

[213] Pangburn MK Rawal N. Structure and function of complement C5 convertase enzymes. Biochem Soc Trans (2002) 30:1006–10. 10.1042/BST0301006 12440962

[214] Rawal N Pangburn MK. Formation of high affinity C5 convertase of the classical pathway of complement. J Biol Chem (2003) 278:38476–83. 10.1074/jbc.M307017200 12878586

[215] Fischer E Kazatchkine MD. Surface-dependent modulation by H of C5 cleavage by the cell-bound alternative pathway C5 convertase of human complement. J Immunol (1983) 1950(130):2821–4. 6222117

[216] Medicus RG Schreiber RD Götze O Müller-Eberhard HJ. A molecular concept of the properdin pathway. Proc Natl Acad Sci U S A (1976) 73:612–6. 10.1073/pnas.73.2.612 54923 PMC335961

[217] Huber-Lang M Sarma JV Zetoune FS Rittirsch D Neff TA McGuire SR Generation of C5a in the absence of C3: a new complement activation pathway. Nat Med (2006) 12:682–7. 10.1038/nm1419 16715088

[218] Amara U Flierl MA Rittirsch D Klos A Chen H Acker B Molecular intercommunication between the complement and coagulation systems. J Immunol (2010) 1950(185):5628–36. 10.4049/jimmunol.0903678 20870944 PMC3123139

[219] Huber-Lang M Younkin EM Sarma JV Riedemann N McGuire SR Lu KT Generation of C5a by phagocytic cells. Am J Pathol (2002) 161:1849–59. 10.1016/S0002-9440(10)64461-6 12414531 PMC1850785

[220] Krisinger MJ Goebeler V Lu Z Meixner SC Myles T Pryzdial ELG Thrombin generates previously unidentified C5 products that support the terminal complement activation pathway. Blood (2012) 120:1717–25. 10.1182/blood-2012-02-412080 22802338

[221] Amara U Rittirsch D Flierl M Bruckner U Klos A Gebhard F Interaction between the coagulation and complement system. Adv Exp Med Biol (2008) 632:71–9. 19025115 10.1007/978-0-387-78952-1_6 PMC2713875

[222] Barthel D Schindler S Zipfel PF. Plasminogen is a complement inhibitor. J Biol Chem (2012) 287:18831–42. 10.1074/jbc.M111.323287 22451663 PMC3365705

[223] Foley JH Peterson EA Lei V Wan LW Krisinger MJ Conway EM. Interplay between fibrinolysis and complement: plasmin cleavage of iC3b modulates immune responses. J Thromb Haemost (2014) 13(4):610–8. 10.1111/jth.12837 25556624

[224] Hadders MA Bubeck D Roversi P Hakobyan S Forneris F Morgan BP Assembly and regulation of the membrane attack complex based on structures of C5b6 and sC5b9. Cell Rep (2012) 1:200–7. 10.1016/j.celrep.2012.02.003 22832194 PMC3314296

[225] Preissner KT Podack ER Müller-Eberhard HJ. The membrane attack complex of complement: relation of C7 to the metastable membrane binding site of the intermediate complex C5b-7. J Immunol (1985) 1950(135):445–51. 3998468

[226] Hadders MA Beringer DX Gros P. Structure of C8alpha-MACPF reveals mechanism of membrane attack in complement immune defense. Science (2007) 317:1552–4. 10.1126/science.1147103 17872444

[227] Bhakdi S Tranum-Jensen J Complement lysis: a hole is a hole. Immunol Today (1991) 12:318–20. 10.1016/0167-5699(91)90007-G 1721819

[228] Cole DS Morgan BP. Beyond lysis: how complement influences cell fate. Clin Sci (2003) 1979(104):455–66. 10.1042/CS20020362 12580763

[229] Koski CL Ramm LE Hammer CH Mayer MM Shin ML. Cytolysis of nucleated cells by complement: cell death displays multi-hit characteristics. Proc Natl Acad Sci U S A (1983) 80:3816–20. 10.1073/pnas.80.12.3816 6602341 PMC394143

[230] Bhakdi S Kuller G Muhly M Fromm S Seibert G Parrisius J. Formation of transmural complement pores in serum-sensitive *Escherichia coli*. Infect Immun (1987) 55:206–10. 3539803 10.1128/iai.55.1.206-210.1987 PMC260303

[231] Lewis LA Ram S Meningococcal disease and the complement system. Virulence (2014) 5:98–126. 10.4161/viru.26515 24104403 PMC3916388

[232] Morgan BP Complement membrane attack on nucleated cells: resistance, recovery and non-lethal effects. Biochem J (1989) 264:1–14. 2690818 10.1042/bj2640001 PMC1133540

[233] Campbell DG Gagnon J Reid KB Williams AF. Rat brain Thy-1 glycoprotein. The amino acid sequence, disulphide bonds and an unusual hydrophobic region. Biochem J (1981) 195:15–30. 6118137 10.1042/bj1950015 PMC1162851

[234] Morgan BP Campbell AK. The recovery of human polymorphonuclear leucocytes from sublytic complement attack is mediated by changes in intracellular free calcium. Biochem J (1985) 231:205–8. 4062885 10.1042/bj2310205 PMC1152725

[235] Nemerow GR Yamamoto KI Lint TF Restriction of complement-mediated membrane damage by the eighth component of complement: a dual role for C8 in the complement attack sequence. J Immunol (1979) 1950(123):1245–52. 469249

[236] Choi NH Mazda T Tomita M. A serum protein SP40,40 modulates the formation of membrane attack complex of complement on erythrocytes. Mol Immunol (1989) 26:835–40. 10.1016/0161-5890(89)90139-9 2601725

[237] Preissner KP Podack ER Müller-Eberhard HJ. SC5b-7, SC5b-8 and SC5b-9 complexes of complement: ultrastructure and localization of the S-protein (vitronectin) within the macromolecules. Eur J Immunol (1989) 19:69–75. 10.1002/eji.1830190112 2465906

[238] Tschopp J Chonn A Hertig S French LE. Clusterin, the human apolipoprotein and complement inhibitor, binds to complement C7, C8 beta, and the b domain of C9. J Immunol (1993) 1950(151):2159–65. 8345200

[239] Fletcher CM Harrison RA Lachmann PJ Neuhaus D. Structure of a soluble, glycosylated form of the human complement regulatory protein CD59. Structure (1994) 1993(2):185–99. 7520819 10.1016/s0969-2126(00)00020-4

[240] Leath KJ Johnson S Roversi P Hughes TR Smith RAG Mackenzie L High-resolution structures of bacterially expressed soluble human CD59. Acta Crystallogr Sect F Struct Biol Cryst Commun (2007) 63:648–52. 10.1107/S1744309107033477 17671359 PMC2335151

[241] Farkas I Baranyi L Ishikawa Y Okada N Bohata C Budai D CD59 blocks not only the insertion of C9 into MAC but inhibits ion channel formation by homologous C5b-8 as well as C5b-9. J Physiol (2002) 539:537–45. 10.1113/jphysiol.2001.013381 11882685 PMC2290142

[242] Meri S Morgan BP Davies A Daniels RH Olavesen MG Waldmann H Human protectin (CD59), an 18,000-20,000 MW complement lysis restricting factor, inhibits C5b-8 catalysed insertion of C9 into lipid bilayers. Immunology (1990) 71:1–9. 1698710 PMC1384213

[243] Huang Y Qiao F Abagyan R Hazard S Tomlinson S. Defining the CD59-C9 binding interaction. J Biol Chem (2006) 281:27398–404. 10.1074/jbc.M603690200 16844690

[244] Lovelace LL Cooper CL Sodetz JM Lebioda L. Structure of human C8 protein provides mechanistic insight into membrane pore formation by complement. J Biol Chem (2011) 286:17585–92. 10.1074/jbc.M111.219766 21454577 PMC3093833

[245] Wickham SE Hotze EM Farrand AJ Polekhina G Nero TL Tomlinson S Mapping the intermedilysin-human CD59 receptor interface reveals a deep correspondence with the binding site on CD59 for complement binding proteins C8alpha and C9. J Biol Chem (2011) 286:20952–62. 10.1074/jbc.M111.237446 21507937 PMC3121471

[246] Bossi F Fischetti F Pellis V Bulla R Ferrero E Mollnes TE Platelet-activating factor and kinin-dependent vascular leakage as a novel functional activity of the soluble terminal complement complex. J Immunol (2004) 1950(173):6921–7. 10.4049/jimmunol.173.11.6921 15557188

[247] Morgan BP Dankert JR Esser AF. Recovery of human neutrophils from complement attack: removal of the membrane attack complex by endocytosis and exocytosis. J Immunol (1987) 1950(138):246–53. 3782799

[248] Moskovich O Fishelson Z. Live cell imaging of outward and inward vesiculation induced by the complement c5b-9 complex. J Biol Chem (2007) 282:29977–86. 10.1074/jbc.M703742200 17644516

[249] Scolding NJ Morgan BP Houston WA Linington C Campbell AK Compston DA. Vesicular removal by oligodendrocytes of membrane attack complexes formed by activated complement. Nature (1989) 339:620–2. 10.1038/339620a0 2733792

[250] Klos A Tenner AJ Johswich K-O Ager RR Reis ES Köhl J. The role of the anaphylatoxins in health and disease. Mol Immunol (2009) 46:2753–66. 10.1016/j.molimm.2009.04.027 19477527 PMC2725201

[251] Aksamit RR Falk W Leonard EJ. Chemotaxis by mouse macrophage cell lines. J Immunol (1981) 1950(126):2194–9. 7229371

[252] Murakami Y Imamichi T Nagasawa S. Characterization of C3a anaphylatoxin receptor on guinea-pig macrophages. Immunology (1993) 79:633–8. 8406589 PMC1421936

[253] Elsner J Oppermann M Czech W Dobos G Schöpf E Norgauer J C3a activates reactive oxygen radical species production and intracellular calcium transients in human eosinophils. Eur J Immunol (1994) 24:518–22. 10.1002/eji.1830240304 8125125

[254] Ehrengruber MU Geiser T Deranleau DA. Activation of human neutrophils by C3a and C5A. Comparison of the effects on shape changes, chemotaxis, secretion, and respiratory burst. FEBS Lett (1994) 346:181–4. 10.1016/0014-5793(94)00463-3 8013630

[255] Elsner J Oppermann M Czech W Kapp A. C3a activates the respiratory burst in human polymorphonuclear neutrophilic leukocytes via pertussis toxin-sensitive G-proteins. Blood (1994) 83:3324–31. 8193368

[256] Coulthard LG Woodruff TM. Is the complement activation product C3a a proinflammatory molecule? Re-evaluating the evidence and the myth. J Immunol (2015) 1950(194):3542–8. 10.4049/jimmunol.1403068 25848071

[257] Kretzschmar T Jeromin A Gietz C Bautsch W Klos A Köhl J Chronic myelogenous leukemia-derived basophilic granulocytes express a functional active receptor for the anaphylatoxin C3a. Eur J Immunol (1993) 23:558–61. 10.1002/eji.1830230239 7679650

[258] Lett-Brown MA Leonard EJ. Histamine-induced inhibition of normal human basophil chemotaxis to C5a. J Immunol (1977) 1950(118):815–8. 845438

[259] el-Lati SG Dahinden CA Church MK. Complement peptides C3a- and C5a-induced mediator release from dissociated human skin mast cells. J Invest Dermatol (1994) 102:803–6. 10.1111/1523-1747.ep12378589 7513741

[260] Nataf S Davoust N Ames RS Barnum SR. Human T cells express the C5a receptor and are chemoattracted to C5a. J Immunol (1999) 1950(162):4018–23. 10201923

[261] Markiewski MM DeAngelis RA Benencia F Ricklin-Lichtsteiner SK Koutoulaki A Gerard C Modulation of the antitumor immune response by complement. Nat Immunol (2008) 9:1225–35. 10.1038/ni.1655 18820683 PMC2678913

[262] Tsuruta T Yamamoto T Matsubara S Nagasawa S Tanase S Tanaka J Novel function of C4a anaphylatoxin. Release from monocytes of protein which inhibits monocyte chemotaxis. Am J Pathol (1993) 142:1848–57. 8506953 PMC1886998

[263] Zhao Y Xu H Yu W Xie B-D. Complement anaphylatoxin C4a inhibits C5a-induced neointima formation following arterial injury. Mol Med Rep (2014) 10:45–52. 10.3892/mmr.2014.2176 24789665 PMC4068717

[264] Barnum SR. C4a: an anaphylatoxin in name only. J Innate Immun (2015). 10.1159/000371423 25659340 PMC6738802

[265] Zhang X Boyar W Toth MJ Wennogle L Gonnella NC. Structural definition of the C5a C terminus by two-dimensional nuclear magnetic resonance spectroscopy. Proteins (1997) 28:261–7. 10.1002/(SICI)1097-0134(199706)28:2<261::AID-PROT13>3.0.CO;2-G 9188742

[266] Matthews KW Mueller-Ortiz SL Wetsel RA. Carboxypeptidase N: a pleiotropic regulator of inflammation. Mol Immunol (2004) 40:785–93. 10.1016/j.molimm.2003.10.002 14687935

[267] Mueller-Ortiz SL Wang D Morales JE Li L Chang J-Y Wetsel RA. Targeted disruption of the gene encoding the murine small subunit of carboxypeptidase N (CPN1) causes susceptibility to C5a anaphylatoxin-mediated shock. J Immunol (2009) 1950(182):6533–9. 10.4049/jimmunol.0804207 19414808 PMC4512742

[268] Bajic G Yatime L Klos A Andersen GR. Human C3a and C3a desArg anaphylatoxins have conserved structures, in contrast to C5a and C5a desArg. Protein Sci (2013) 22:204–12. 10.1002/pro.2200 23184394 PMC3588916

[269] Cook WJ Galakatos N Boyar WC Walter RL Ealick SE. Structure of human desArg-C5a. Acta Crystallogr D Biol Crystallogr (2010) 66:190–7. 10.1107/S0907444909049051 20124699

[270] Sayah S Jauneau AC Patte C Tonon MC Vaudry H Fontaine M. Two different transduction pathways are activated by C3a and C5a anaphylatoxins on astrocytes. Brain Res Mol Brain Res (2003) 112:53–60. 10.1016/S0169-328X(03)00046-9 12670702

[271] Schatz-Jakobsen JA Yatime L Larsen C Petersen SV Klos A Andersen GR. Structural and functional characterization of human and murine C5a anaphylatoxins. Acta Crystallogr D Biol Crystallogr (2014) 70:1704–17. 10.1107/S139900471400844X 24914981 PMC4051506

[272] Gao J Choe H Bota D Wright PL Gerard C Gerard NP. Sulfation of tyrosine 174 in the human C3a receptor is essential for binding of C3a anaphylatoxin. J Biol Chem (2013) 278:37902–8. 10.1074/jbc.M306061200 12871936

[273] Venkatesha RT Berla Thangam E Zaidi AK Ali H. Distinct regulation of C3a-induced MCP-1/CCL2 and RANTES/CCL5 production in human mast cells by extracellular signal regulated kinase and PI3 kinase. Mol Immunol (2005) 42:581–7. 10.1016/j.molimm.2004.09.009 15607817

[274] Mery L Boulay F. Evidence that the extracellular N-terminal domain of C5aR contains amino-acid residues crucial for C5a binding. Eur J Haematol (1993) 51:282–7. 10.1111/j.1600-0609.1993.tb01609.x 8282089

[275] Siciliano SJ Rollins TE DeMartino J Konteatis Z Malkowitz L Van Riper G Two-site binding of C5a by its receptor: an alternative binding paradigm for G protein-coupled receptors. Proc Natl Acad Sci U S A (1994) 91:1214–8. 10.1073/pnas.91.4.1214 8108389 PMC43127

[276] Huber-Lang MS Sarma JV McGuire SR Lu KT Padgaonkar VA Younkin EM Structure-function relationships of human C5a and C5aR. J Immunol (2003) 1950(170):6115–24. 10.4049/jimmunol.170.12.6115 12794141

[277] Gerber BO Meng EC Dotsch V Baranski TJ Bourne HR. An activation switch in the ligand binding pocket of the C5a receptor. J Biol Chem (2001) 276:3394–400. 10.1074/jbc.M007748200 11062244

[278] Matsumoto ML Narzinski K Kiser PD Nikiforovich GV Baranski TJ. A comprehensive structure-function map of the intracellular surface of the human C5a receptor. I. Identification of critical residues. J Biol Chem (2007) 282:3105–21. 10.1074/jbc.M607679200 17135254

[279] Klco JM Wiegand CB Narzinski K Baranski TJ. Essential role for the second extracellular loop in C5a receptor activation. Nat Struct Mol Biol (2005) 12:320–6. 10.1038/nsmb913 15768031

[280] Perianayagam MC Balakrishnan VS King AJ Pereira BJG Jaber BL. C5a delays apoptosis of human neutrophils by a phosphatidylinositol 3-kinase-signaling pathway. Kidney Int (2002) 61:456–63. 10.1046/j.1523-1755.2002.00139.x 11849385

[281] la Sala A Gadina M Kelsall BL. G(i)-protein-dependent inhibition of IL-12 production is mediated by activation of the phosphatidylinositol 3-kinase-protein 3 kinase B/Akt pathway and JNK. J Immunol (2005) 1950(175):2994–9. 10.4049/jimmunol.175.5.2994 16116186

[282] Jiang H Kuang Y Wu Y Xie W Simon MI Wu D. Roles of phospholipase C beta2 in chemoattractant-elicited responses. Proc Natl Acad Sci U S A (1997) 94:7971–5. 10.1073/pnas.94.15.7971 9223297 PMC21539

[283] Mullmann TJ Siegel MI Egan RW Billah MM. Complement C5a activation of phospholipase D in human neutrophils. A major route to the production of phosphatidates and diglycerides. J Immunol (1990) 1950(144):1901–8. 2307846

[284] Buhl AM Avdi N Worthen GS Johnson GL. Mapping of the C5a receptor signal transduction network in human neutrophils. Proc Natl Acad Sci U S A (1994) 91:9190–4. 10.1073/pnas.91.19.9190 8090790 PMC44773

[285] Lee DK George SR Cheng R Nguyen T Liu Y Brown M Identification of four novel human G protein-coupled receptors expressed in the brain. Brain Res Mol Brain Res (2001) 86:13–22. 10.1016/S0169-328X(00)00242-4 11165367

[286] Okinaga S Slattery D Humbles A Zsengeller Z Morteau O Kinrade MB C5L2, a nonsignaling C5A binding protein. Biochemistry (2003) 42:9406–15. 10.1021/bi034489v 12899627

[287] Cain SA Monk PN. The orphan receptor C5L2 has high affinity binding sites for complement fragments C5a and C5a des-Arg(74). J Biol Chem (2002) 277:7165–9. 10.1074/jbc.C100714200 11773063

[288] Wang R Lu B Gerard C Gerard NP. Disruption of the complement anaphylatoxin receptor C5L2 exacerbates inflammation in allergic contact dermatitis. J Immunol (2013) 1950(191):4001–9. 10.4049/jimmunol.1301626 24043888 PMC4551435

[289] Poursharifi P Lapointe M Pétrin D Devost D Gauvreau D Hébert TE C5L2 and C5aR interaction in adipocytes and macrophages: insights into adipoimmunology. Cell Signal (2013) 25:910–8. 10.1016/j.cellsig.2012.12.010 23268185

[290] Hsu W-C Yang F-C Lin C-H Hsieh S-L Chen N-J. C5L2 is required for C5a-triggered receptor internalization and ERK signaling. Cell Signal (2014) 26:1409–19. 10.1016/j.cellsig.2014.02.021 24631530

[291] DeFea KA Zalevsky J Thoma MS Déry O Mullins RD Bunnett NW. beta-arrestin-dependent endocytosis of proteinase-activated receptor 2 is required for intracellular targeting of activated ERK1/2. J Cell Biol (2000) 148:1267–81. 10.1083/jcb.148.6.1267 10725339 PMC2174299

[292] DeWire SM Ahn S Lefkowitz RJ Shenoy SK. **β**-arrestins and cell signaling. Annu Rev Physiol (2007) 69:483–510. 10.1146/annurev.physiol.69.022405.154749 17305471

[293] van Lookeren Campagne M Wiesmann C Brown EJ. Macrophage complement receptors and pathogen clearance. Cell Microbiol (2007) 9:2095–102. 10.1111/j.1462-5822.2007.00981.x 17590164

[294] He JQ Wiesmann C van Lookeren Campagne M. A role of macrophage complement receptor CRIg in immune clearance and inflammation. Mol Immunol (2008) 45:4041–7. 10.1016/j.molimm.2008.07.011 18752851

[295] Vogt L Schmitz N Kurrer MO Bauer M Hinton HI Behnke S VSIG4, a B7 family-related protein, is a negative regulator of T cell activation. J Clin Invest (2006) 116:2817–26. 10.1172/JCI25673 17016562 PMC1578631

[296] Matsumoto AK Martin DR Carter RH Klickstein LB Ahearn JM Fearon DT. Functional dissection of the CD21/CD19/TAPA-1/Leu-13 complex of B lymphocytes. J Exp Med (1993) 178:1407–17. 10.1084/jem.178.4.1407 7690834 PMC2191213

[297] Dempsey PW Allison ME Akkaraju S Goodnow CC Fearon DT. C3d of complement as a molecular adjuvant: bridging innate and acquired immunity. Science (1996) 271:348–50. 10.1126/science.271.5247.348 8553069

[298] Carroll MC Isenman DE. Regulation of humoral immunity by complement. Immunity (2012) 37:199–207. 10.1016/j.immuni.2012.08.002 22921118 PMC5784422

[299] Kalli KR Ahearn JM Fearon DT. Interaction of iC3b with recombinant isotypic and chimeric forms of CR2. J Immunol (1991) 1950(147):590–4. 1830068

[300] Szakonyi G Guthridge JM Li D Young K Holers VM Chen XS. Structure of complement receptor 2 in complex with its C3d ligand. Science (2001) 292:1725–8. 10.1126/science.1059118 11387479

[301] Hannan JP Young KA Guthridge JM Asokan R Szakonyi G Chen XS Mutational analysis of the complement receptor type 2 (CR2/CD21)-C3d interaction reveals a putative charged SCR1 binding site for C3d. J Mol Biol (2005) 346:845–58. 10.1016/j.jmb.2004.12.007 15713467

[302] Isenman DE Leung E Mackay JD Bagby S van den Elsen JMH Mutational analyses reveal that the staphylococcal immune evasion molecule Sbi and complement receptor 2 (CR2) share overlapping contact residues on C3d: implications for the controversy regarding the CR2/C3d cocrystal structure. J Immunol (2010) 1950(184):1946–55. 10.4049/jimmunol.0902919 20083651

[303] Springer TA Dustin ML. Integrin inside-out signaling and the immunological synapse. Curr Opin Cell Biol (2012) 24:107–15. 10.1016/j.ceb.2011.10.004 22129583 PMC3294052

[304] Underhill DM Ozinsky A. Phagocytosis of microbes: complexity in action. Annu Rev Immunol (2002) 20:825–52. 10.1146/annurev.immunol.20.103001.114744 11861619

[305] Bajic G Yatime L Sim RB Vorup-Jensen T Andersen GR. Structural insight on the recognition of surface-bound opsonins by the integrin I domain of complement receptor 3. Proc Natl Acad Sci U S A (2013) 110:16426–31. 10.1073/pnas.1311261110 24065820 PMC3799375

[306] Chen X Yu Y Mi L-Z Walz T Springer TA. Molecular basis for complement recognition by integrin **α**X**β**2. Proc Natl Acad Sci U S A (2012) 109:4586–91. 10.1073/pnas.1202051109 22393018 PMC3311339

[307] Diamond MS Garcia-Aguilar J Bickford JK Corbi AL Springer TA. The I domain is a major recognition site on the leukocyte integrin Mac-1 (CD11b/CD18) for four distinct adhesion ligands. J Cell Biol (1993) 120:1031–43. 10.1083/jcb.120.4.1031 7679388 PMC2200080

[308] Alcorlo M Martínez-Barricarte R Fernández FJ Rodríguez-Gallego C Round A Vega MC Unique structure of iC3b resolved at a resolution of 24 Å by 3D-electron microscopy. Proc Natl Acad Sci U S A (2011) 108:13236–40. 10.1073/pnas.1106746108 21788512 PMC3156172

[309] Sen M Yuki K Springer TA. An internal ligand-bound, metastable state of a leukocyte integrin, **α**X**β**2. J Cell Biol (2013) 203:629–42. 10.1083/jcb.201308083 24385486 PMC3840939

[310] Gorgani NN He JQ Katschke KJ Helmy KY Xi H Steffek M Complement receptor of the Ig superfamily enhances complement-mediated phagocytosis in a subpopulation of tissue resident macrophages. J Immunol (2008) 1950(181):7902–8. 10.4049/jimmunol.181.11.7902 19017980

[311] Helmy KY Katschke KJ Gorgani NN Kljavin NM Elliott JM Diehl L CRIg: a macrophage complement receptor required for phagocytosis of circulating pathogens. Cell (2006) 124:915–27. 10.1016/j.cell.2005.12.039 16530040

[312] Wiesmann C Katschke KJ Yin J Helmy KY Steffek M Fairbrother WJ Structure of C3b in complex with CRIg gives insights into regulation of complement activation. Nature (2006) 444:217–20. 10.1038/nature05263 17051150

[313] Malina M Roumenina LT Seeman T Le Quintrec M Dragon-Durey M-A Schaefer F Genetics of hemolytic uremic syndromes. Presse Méd (2012) 1983(41):e105–14. 10.1016/j.lpm.2011.10.028 22265161

[314] Noris M Remuzzi G Atypical hemolytic-uremic syndrome. N Engl J Med (2009) 361:1676–87. 10.1056/NEJMra0902814 19846853

[315] Roumenina LT Loirat C Dragon-Durey M-A Halbwachs-Mecarelli L Sautes-Fridman C Fremeaux-Bacchi V. Alternative complement pathway assessment in patients with atypical HUS. J Immunol Methods (2011) 365:8–26. 10.1016/j.jim.2010.12.020 21215749

[316] Manuelian T Hellwage J Meri S Caprioli J Noris M Heinen S Mutations in factor H reduce binding affinity to C3b and heparin and surface attachment to endothelial cells in hemolytic uremic syndrome. J Clin Invest (2003) 111:1181–90. 10.1172/JCI200316651 12697737 PMC152934

[317] Sánchez-Corral P Pérez-Caballero D Huarte O Simckes AM Goicoechea E López-Trascasa M Structural and functional characterization of factor H mutations associated with atypical hemolytic uremic syndrome. Am J Hum Genet (2002) 71:1285–95. 10.1086/344515 12424708 PMC378565

[318] Sánchez-Corral P González-Rubio C Rodríguez de Córdoba S López-Trascasa M. Functional analysis in serum from atypical hemolytic uremic syndrome patients reveals impaired protection of host cells associated with mutations in factor H. Mol Immunol (2004) 41:81–4. 10.1016/j.molimm.2004.01.003 15140578

[319] Frémeaux-Bacchi V Miller EC Liszewski MK Strain L Blouin J Brown AL Mutations in complement C3 predispose to development of atypical hemolytic uremic syndrome. Blood (2008) 112:4948–52. 10.1182/blood-2008-01-133702 18796626 PMC2597601

[320] Roumenina LT Frimat M Miller EC Provot F Dragon-Durey M-A Bordereau P A prevalent C3 mutation in aHUS patients causes a direct C3 convertase gain of function. Blood (2012) 119:4182–91. 10.1182/blood-2011-10-383281 22246034 PMC3359738

[321] Marinozzi MC Vergoz L Rybkine T Ngo S Bettoni S Pashov A Complement factor B mutations in atypical hemolytic uremic syndrome-disease-relevant or benign? J Am Soc Nephrol (2014) 25:2053–65. 10.1681/ASN.2013070796 24652797 PMC4147975

[322] Goicoechea de Jorge E Harris CL Esparza-Gordillo J Carreras L Arranz EA Garrido CA Gain-of-function mutations in complement factor B are associated with atypical hemolytic uremic syndrome. Proc Natl Acad Sci U S A (2007) 104:240–5. 10.1073/pnas.0603420103 17182750 PMC1765442

[323] Roumenina LT Jablonski M Hue C Blouin J Dimitrov JD Dragon-Durey M-A Hyperfunctional C3 convertase leads to complement deposition on endothelial cells and contributes to atypical hemolytic uremic syndrome. Blood (2009) 114:2837–45. 10.1182/blood-2009-01-197640 19584399

[324] Ricklin D Lambris JD. Complement in immune and inflammatory disorders: therapeutic interventions. J Immunol (2013) 1950(190):3839–47. 10.4049/jimmunol.1203200 23564578 PMC3623010

[325] Roumenina LT Zuber J Frémeaux-Bacchi V. Physiological and therapeutic complement regulators in kidney transplantation. Curr Opin Organ Transplant (2013) 18:421–9. 10.1097/MOT.0b013e32836370ce 23838647

[326] Rother RP Rollins SA Mojcik CF Brodsky RA Bell L. Discovery and development of the complement inhibitor eculizumab for the treatment of paroxysmal nocturnal hemoglobinuria. Nat Biotechnol (2007) 25:1256–64. 10.1038/nbt1344 17989688

[327] Hillmen P Young NS Schubert J Brodsky RA Socié G Muus P The complement inhibitor eculizumab in paroxysmal nocturnal hemoglobinuria. N Engl J Med (2006) 355:1233–43. 10.1056/NEJMoa061648 16990386

[328] Legendre CM Licht C Loirat C Eculizumab in atypical hemolytic-uremic syndrome. N Engl J Med (2013) 369:1379–80. 10.1056/NEJMc1308826 24088105

[329] Zuber J Fakhouri F Roumenina LT Loirat C Frémeaux-Bacchi V French Study Group for aHUS/C3G. Use of eculizumab for atypical haemolytic uraemic syndrome and C3 glomerulopathies. Nat Rev Nephrol (2012) 8:643–57. 10.1038/nrneph.2012.214 23026949

[330] Morikis D Assa-Munt N Sahu A Lambris JD Solution structure of compstatin, a potent complement inhibitor. Protein Sci (1998) 7:619–27. 10.1002/pro.5560070311 9541394 PMC2143948

[331] Ricklin D Lambris JD. Compstatin: a complement inhibitor on its way to clinical application. Adv Exp Med Biol (2008) 632:273–92. 19025129 10.1007/978-0-387-78952-1_20 PMC2700864

[332] Janssen BJC Halff EF Lambris JD Gros P. Structure of compstatin in complex with complement component C3c reveals a new mechanism of complement inhibition. J Biol Chem (2007) 282:29241–7. 10.1074/jbc.M704587200 17684013

[333] Nilsson B Larsson R Hong J Elgue G Ekdahl KN Sahu A Compstatin inhibits complement and cellular activation in whole blood in two models of extracorporeal circulation. Blood (1998) 92:1661–7. 9716594

[334] Silasi-Mansat R Zhu H Popescu NI Peer G Sfyroera G Magotti P Complement inhibition decreases the procoagulant response and confers organ protection in a baboon model of *Escherichia coli* sepsis. Blood (2010) 116:1002–10. 10.1182/blood-2010-02-269746 20466856 PMC2924221

[335] Risitano AM Ricklin D Huang Y Reis ES Chen H Ricci P Peptide inhibitors of C3 activation as a novel strategy of complement inhibition for the treatment of paroxysmal nocturnal hemoglobinuria. Blood (2014) 123:2094–101. 10.1182/blood-2013-11-536573 24497537 PMC3968392

[336] Hebecker M Alba-Domínguez M Roumenina LT Reuter S Hyvärinen S Dragon-Durey M-A An engineered construct combining complement regulatory and surface-recognition domains represents a minimal-size functional factor H. J Immunol (2013) 1950(191):912–21. 10.4049/jimmunol.1300269 23772024

[337] Schmidt CQ Bai H Lin Z Risitano AM Barlow PN Ricklin D Rational engineering of a minimized immune inhibitor with unique triple-targeting properties. J Immunol (2013) 1950(190):5712–21. 10.4049/jimmunol.1203548 23616575 PMC3825029

[338] Fridkis-Hareli M Storek M Mazsaroff I Risitano AM Lundberg AS Horvath CJ Design and development of TT30, a novel C3d-targeted C3/C5 convertase inhibitor for treatment of human complement alternative pathway-mediated diseases. Blood (2011) 118:4705–13. 10.1182/blood-2011-06-359646 21860027 PMC3208285

[339] Li K Gor J Holers VM Storek MJ Perkins SJ. Solution structure of TT30, a novel complement therapeutic agent, provides insight into its joint binding to complement C3b and C3d. J Mol Biol (2012) 418:248–63. 10.1016/j.jmb.2012.02.038 22387467

[340] Huang Y Qiao F Atkinson C Holers VM Tomlinson S. A novel targeted inhibitor of the alternative pathway of complement and its therapeutic application in ischemia/reperfusion injury. J Immunol (2008) 1950(181):8068–76. 10.4049/jimmunol.181.11.8068 19017999 PMC2782395

[341] Risitano AM Notaro R Pascariello C Sica M del Vecchio L Horvath CJ The complement receptor 2/factor H fusion protein TT30 protects paroxysmal nocturnal hemoglobinuria erythrocytes from complement-mediated hemolysis and C3 fragment. Blood (2012) 119:6307–16. 10.1182/blood-2011-12-398792 22577173

[342] Rohrer B Long Q Coughlin B Renner B Huang Y Kunchithapautham K A targeted inhibitor of the complement alternative pathway reduces RPE injury and angiogenesis in models of age-related macular degeneration. Adv Exp Med Biol (2010) 703:137–49. 10.1007/978-1-4419-5635-4_10 20711712

